# Artificial Intelligence in Pancreatic Image Analysis: A Review

**DOI:** 10.3390/s24144749

**Published:** 2024-07-22

**Authors:** Weixuan Liu, Bairui Zhang, Tao Liu, Juntao Jiang, Yong Liu

**Affiliations:** 1 Sydney Smart Technology College, Northeastern University at Qinhuangdao, Qinhuangdao 066004, China; weixuanliu@stumail.neu.edu.cn (W.L.); 202119037@stu.neu.edu.cn (B.Z.); 2 School of Mathematics and Statistics, Northeastern University at Qinhuangdao, Qinhuangdao 066004, China; liutao@neuq.edu.cn; 3 College of Control Science and Engineering, Zhejiang University, Hangzhou 310058, China

**Keywords:** pancreatic cancer, artificial intelligence, medical images, diagnosis, treatment

## Abstract

Pancreatic cancer is a highly lethal disease with a poor prognosis. Its early diagnosis and accurate treatment mainly rely on medical imaging, so accurate medical image analysis is especially vital for pancreatic cancer patients. However, medical image analysis of pancreatic cancer is facing challenges due to ambiguous symptoms, high misdiagnosis rates, and significant financial costs. Artificial intelligence (AI) offers a promising solution by relieving medical personnel’s workload, improving clinical decision-making, and reducing patient costs. This study focuses on AI applications such as segmentation, classification, object detection, and prognosis prediction across five types of medical imaging: CT, MRI, EUS, PET, and pathological images, as well as integrating these imaging modalities to boost diagnostic accuracy and treatment efficiency. In addition, this study discusses current hot topics and future directions aimed at overcoming the challenges in AI-enabled automated pancreatic cancer diagnosis algorithms.

## 1. Introduction

Pancreatic cancer (PC) is a lethal digestive tumor with a very poor prognosis. Its symptoms are often mild until advanced stages, and it tends to recur after surgical removal [1]. Its mortality and morbidity are highly paralleled and pose a great threat to human health [2]. Pancreatic ductal adenocarcinoma (PDAC) is the most common type of PC, 80–85% of which present with advanced local or distant metastatic disease, while only 15–20% is suitable for surgical removal. In addition, the 5-year relative survival rate for PC is 12%, the lowest among all types of cancers [3]. These data suggest that early screening and diagnosis are important in improving survival outcomes in patients with PC.

Medical imaging techniques are becoming increasingly important for PCs, as they provide tissue information and could be used for diagnosis, treatment determination, and prognosis monitoring [4]. Current advanced medical imaging tools primarily include computed tomography (CT), magnetic resonance imaging (MRI), endoscopic ultrasound (EUS), positron emission tomography (PET), and pathological images [5,6]. Improvements based on these imaging tools include EUS-guided fine-needle aspiration (FNA) and biopsy (FNB), contrast-enhanced EUS (CE-EUS), contrast-enhanced computed tomography (CE-CT), contrast-enhanced magnetic resonance imaging (CE-MRI), and positron emission tomography (PET/CT).

The above-mentioned imaging tools have advantages and disadvantages. CT is the most commonly used tool that acquires tomographic images of the body through X-rays [7]. At the same time, its resolution for small and variable organs like the pancreas is limited. EUS has a higher resolution but is complicated to operate and the field of view is narrow. MRI generates soft-tissue imaging and can better distinguish between tumor and normal tissues, but has a longer time and higher cost. PET reflects tumor metabolism that assesses PC metastasis, but has lower resolution and is usually combined with CT. Pathological imaging is an invasive way of slicing and staining tissue samples. Although there are now multiple medical imaging modalities available, some early PCs will not be detected by CT, MRI, or EUS [8]. Manual diagnosis based on currently available imaging techniques is insufficient. The accurate diagnosis of PC still relies highly on invasive biopsies after the imaging step, which is complex and time-consuming. This delay may result in patients missing critical treatment opportunities.

In recent years, the AI-powered image process has been applied to the diagnosis of PC in the experimental stage with reasonable results, marking the beginning of a shift from the traditional diagnosis dependent on biopsy. AI is a computer technology that can simulate specific human behaviors, such as learning, reasoning, problem-solving, and decision-making. Deep learning is a milestone of AI and utilizes propagation algorithms, which have made significant breakthroughs in automated image analysis with high Accuracy, Specificity, and Recall in diagnosing PC and differentiating it from chronic pancreatitis [9,10]. Well-trained AI models can process input medical images and output analytical results within seconds, minimizing the trauma to the patient. Studies have shown that AI models report comparable results with medical experts in PC detection and even better results in some cases [11,12,13,14]. Their significant cost and speed advantages also improve clinical diagnosis, treatment, and prognosis of PC, reducing the workload of doctors and the financial burden of patients.

For the diagnosis stage, the low prevalence of PC leads to a lack of early screening. Fast, low-cost AI models facilitate the scaling up of medical image-based early screening. Small and subtle lesions or precursors that might be missed by traditional diagnostic methods could be detected. As a result, more potential PC patients could notice their health condition in time and be operated on as soon as possible. The PC metastasis rate and mortality will be reduced. For the treatment stage, AI models could predict PC metastasis and the survival time of patients after surgery using image information [14,15]. Since the tissue structure of PC is complex and targeted therapies are insufficient for high costs, AI could help doctors make appropriate treatment decisions and reduce overall costs. Rational and timely treatment strategies in turn improve the prognostic outcome of PCs. Studies have shown that, in clinical trials, AI can reduce the burden of routine tasks in the medical workflow, allowing doctors to spend more time tackling other challenges [16]. Therefore, the automated analysis of pancreatic images by AI is an efficient and convenient aid to doctors.

### 1.1. Contribution of This Review

This paper is a comprehensive review of the application of various AI models to medical images in five modalities of pancreatic images. Sub-types of pancreatic cancer, evaluation metrics for different tasks, and mainstream AI models are discussed. The main contributions are as follows:

- There is a brief description of PC, including its characteristics, subtypes, risk factors, precursor lesions, and clinical challenges.
- There is a summary of the various AI tasks, representative models for each task, and the metrics used to evaluate the performance of AI models on each task.
- There is an outline of publicly available pancreatic image datasets with different modalities and comparisons of AI model performance on some of them.
- This paper describes the imaging features of CT, MRI, EUS, PET, pathological images, and their combination. It also comprehensively discusses the application of AI models in pancreatic medical image analysis for different tasks on different modalities.
- This paper summarizes visualization tools, deep learning frameworks, and software for processing and analyzing pancreatic images.
- This paper also discusses current clinical challenges and future research directions for AI models to improve the outcomes of PC diagnosis and treatment.

Some reviews have discussed the potential and effectiveness of combining AI techniques and pancreatic images. Cazacu et al. [17] surveyed deep learning models, mainly CNNs, to differentiate PC and chronic pancreatitis (CP) on EUS images and validated their high performance in diagnosis. Pereira et al. [18] examined AI in CT and MRI images for PDAC early detection and prognosis evaluation. Kenner et al. [19] discussed the early detection of PDAC, the application of AI models to PDAC, the organizational structure to screen for PDAC, and the reflections of government, industry and advocacy. Yang et al. [20] outlined early PC screening and diagnosis approaches, such as imaging, pathological examination, serological examination, and liquid biopsy, with AI recognized as an innovative potential strategy. Huang et al. [16] summarized AI applications in medical image analysis, pathological examination, and biomarkers in PC diagnosis; survival time, risk of recurrence, metastasis, and response to therapy in PC prognosis. Both limitations and significant potential of AI were identified in their work. Hameed and Krishnan [21] explored the AI-enabled PC diagnosis on four imaging modalities (EUS, MRI, CT, and PET), cytopathology, and serological markers. Ethical concerns about AI tools were also noted. Schlanger et al. [22] discussed AI and machine learning models for three PC surgery stages: preoperative diagnosis, intraoperative complication prediction, and prognostic evaluation. Their findings suggested that, while AI demonstrated great potential in diagnosis and prognosis, its research on intraoperative applications was still limited. Mikdadi et al. [23] detailed the advancements of AI in PDAC diagnosis and prognosis from CT images. Jan et al. [24] synthesized AI techniques in PC prediction and early diagnosis, including AI tasks, models, medical data types, programming languages, and validation approaches. They noted that future PDAC detection could rely on a suite of models for whole-body regions rather than specific organs. Katta et al. [25] reviewed AI in PC biomarkers detection, diagnosis, and prognosis. They also identified shortcomings of AI applications in knowledge, data processing, ethics, and clinical implementation. Zhao et al. [26] summarized AI in early screening, diagnosis, surgical treatment, and prognostic prediction. They also identified potential dividends in the future despite current limitations of AI in terms of interpretability, generalizability, sample size, and ethical concerns. Daher et al. [27] delved into machine learning and deep learning approaches in PC detection based on CT, EUS, MRI, and PET images and their ethical concerns.

Table 1 compares this paper and existing reviews on AI-enabled pancreatic image processing. H (high) means that there is an in-depth discussion on this topic in the review article. M (moderate) represents that the article contained a chapter or paragraph containing that topic. L (low) implies the article mentioned that topic, but lacked sufficient explanation. N (none) indicates that the article did not cover the topic. Compared with existing relevant reviews, our work covers a wider range of topics and discusses them more in depth.

### 1.2. Structure of This Review

This paper aims to discuss the current status and future direction of AI applications in pancreatic image processing. By summarizing the AI methods for detecting PCs, it provides an effective reference for PC early screening and diagnostic solutions and promotes potential transformations in the field of medical diagnosis. Section 2 provides an overview of the databases used, literature search methods, and selection strategies. Section 3 discusses the subtypes of PC, its challenges in clinical diagnosis and treatment, and the significance of AI applications. Section 4 reviews the currently publicly available pancreatic medical imaging datasets. Section 5 outlines different AI tasks, representative models, and evaluation metrics for the performance of the models on these tasks. Section 6, Section 7, Section 8, Section 9 and Section 10 summarize the application of AI models on CT, MRI, EUS, PET, and pathological images, respectively. Section 11 reviews the application of AI models combining multiple image modalities. Section 12 lists the software, frameworks, and tools for analyzing medical image data. Section 13 points out future research topics.

## 2. Materials and Methods

### 2.1. Search Strategy and Literature Sources

We follow the PRISMA guidelines [28] (preferred reporting items for systematic reviews and meta-analyses) and search for relevant articles through repositories and databases such as IEEE, ScienceDirect, PubMed, Web of Science, Scopus, etc. Table 2 shows sets of keywords associated with terms used to search the literature, including pancreatic cancer, cancer diagnosis, AI task, deep learning, machine learning, etc. A total of 370 nonduplicated articles were initially screened based on these keywords.

### 2.2. Selection Criteria

Each article initially screened was confirmed by two authors for inclusion, with a third author deciding if there was disagreement. Articles published within the last two decades (2004–2024) were included. Research quality, information completeness, journal authority, citation number, relevancy, and redundancy were considered in the elimination process.

### 2.3. Results

**Figure 1:**
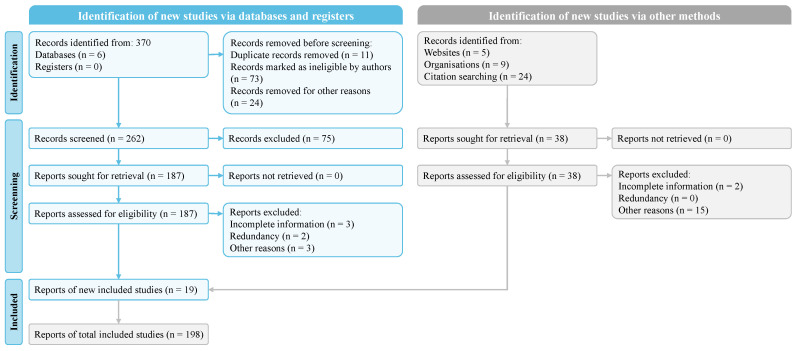
PRISMA flowchart.

Of the 370 articles initially screened from various databases, 108 were excluded by screening the titles and abstracts. In addition, 75 articles were excluded by full-text analysis due to research quality, incomplete information, journal reputation, redundancy, etc. In addition, 19 additional reports were added when we reviewed the references of the selected articles, websites of public datasets, and relevant organizations. In the end, 198 reports were used to develop this review based on the selection criteria. Figure 1 shows the PRISMA implementation process.

## 3. Pancreatic Cancer and Clinical Challenges

### 3.1. Introduction to Pancreatic Cancer

The pancreas consists of the head, neck, body and tail of the pancreas, located in the abdominal cavity. PC is usually referred to as a tumor that arises within the epithelial cells of the pancreas [29]. Smoking, obesity, diabetes mellitus, alcohol, pancreatitis, allergies, the microbiome, the environment, occupation, family history of cancer, and CP are risk factors of PCs [30,31]. Autoimmune pancreatitis (AIP) is a rare form of CP [31]. Pancreatic intraepithelial neoplasms (PanINs), intraductal papillary mucinous neoplasms (IPMNs), and mucinous cystic neoplasms (MCNs) are precursor lesions of PC [32].

**Figure 2:**
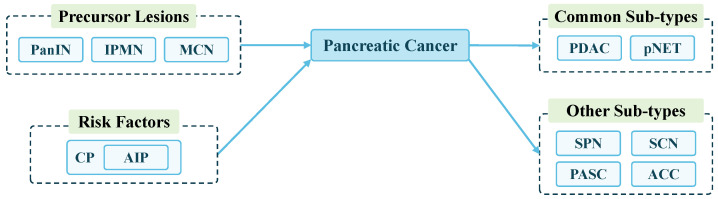
Precursors, risk factors, and subtypes of PC.

PC presents in two common types: pancreatic ductal adenocarcinoma (PDAC) and pancreatic neuroendocrine tumor (pNET) [33]. PDAC accounts for more than 90% of PCs and is the most prevalent type, while pNET is relatively rare, accounting for less than 5% [34]. Among other rare types, solid pseudopapillary neoplasm (SPN) represents 0.2–2.7% of PCs, which typically affects young females [35]. Additional infrequent types of PC include serous cystic neoplasms (SCN), pancreatic adenosquamous carcinoma (PASC), acinar cell carcinoma (ACC), etc. [36,37,38,39]. Due to their rarity, these tumors lack large-scale clinical studies, and many issues remain unclear. Using AI to differentiate between these tumors can help doctors learn their characteristics. Figure 2 shows the relationship between these lesions.

#### 3.1.1. Pancreatic Ductal Adenocarcinoma

Pancreatic ductal adenocarcinoma (PDAC) is a malignancy affecting the exocrine pancreas and involving acinar and duct cells with a contentious origin. Although traditionally believed to originate from duct cells, studies in rodents have suggested an alternative origin from acinar cells [40]. The development of PDAC typically commences with pancreatic intraepithelial neoplasias, marked by the accrual of genetic mutations. Some cases of PDAC are attributed to these precursor lesions, such as IPMN [1]. According to statistical analysis on data from the SEER cancer registry [41], PDAC located in the body and tail of the pancreas is associated with worse survival than PDAC located in the head of the pancreas [42]. As PDAC advances, its potential for extensive spread becomes pronounced once it reaches a critical size at its primary site, underscoring the aggressive nature of this malignancy [43]. According to the gene expression profiles of malignant epithelial cells, PDAC can be divided into subtypes [44]. In the two-group classification, the main subtypes of PDAC include the classical subtype and the basal-like subtype. The basal-like subtype has been associated with a poorer prognosis and a more aggressive phenotype [45,46,47].

#### 3.1.2. Pancreatic Neuroendocrine Tumors

Pancreatic neuroendocrine tumor (pNET) is a rare and diverse neoplasm. According to population studies, the incidence of pNET is less than 1 in 100,000 [48]. However, with increasing use of CT scans, the incidence has doubled in the last few decades [49]. pNET arises from pancreatic neuroendocrine cells. They are found in various organs and are vital for receiving signals from the nervous system and regulating numerous bodily functions. pNET can be divided into functional pNET (F-pNET), which secrete hormones leading to specific clinical syndromes, and non-functional pNET (NF-pNET), which have no symptoms [50,51,52]. F-pNET are relatively rare and account for about 20%. The most common F-pNET are insulinomas, which lead to hypoglycemia, and gastrinomas, which lead to excessive gastrin overproduction. Other less common types include glucagonomas, VIPomas, and somatostatinomas [53,54]. F-pNET exhibits varying degrees of malignant potential across subtypes. Specifically, the insulinomas subtype is the most benign one with a malignant potential of 5 to 15%, while other subtypes have a much higher potential ranging from 60 to 90% [55]. NF-pNET can be divided into three categories: those that do not produce hormones; those that have hormones at levels low enough to cause symptoms; and those that produce hormones like pancreatic polypeptide, chromogranin A, ghrelin, calcitonin, or neurotensin that do not cause symptoms [56]. Compared with F-pNET, NF-pNET is typically discovered later and is more prone to malignancy, often leading to poorer prognoses [57]. These types of tumors generally remain asymptomatic until they reach a substantial size, at which point symptoms emerge due to the mass effects of the original tumor or its metastasis [56]. Furthermore, pNET tends to be multifocal and can metastasize to other organs, with the liver being the primary site of metastasis, significantly impacting the overall prognosis [58,59].

### 3.2. Clinical Challenges of PC Diagnosis and Treatment

Accurately diagnosing PC poses significant challenges. While screening for early cancer precursors and the subsequent surgical removal of diseased lesions can reduce morbidity and mortality [60], the relatively low incidence of PC among diseases makes screening for asymptomatic individuals unfeasible [18]. Moreover, symptoms in patients with early-stage PC are typically mild and can be mistaken for common benign diseases [61]. As a result, most PCs are diagnosed after metastasis has occurred, with only a small number being identified at the local stage. Unfortunately, poor survival rates have not significantly improved in recent decades [62]. Compounding this issue, the lack of knowledge about PC and diagnostic pathways often results in patients reluctant to seek medical attention, causing treatment delays [63]. Additionally, existing diagnostic methods exhibit a high false positive rate and lack effectiveness [18]. The heterogeneous behavior of PC further complicates matters, as it can be challenging to determine malignant potential accurately, and overdiagnosis can potentially do more harm than good in mortality [60]. AI models are efficient and fast to detect PCs; therefore, they can reduce the cost of screening and the incidence of misdiagnosis.

The treatment of PC also presents significant challenges. Currently, the primary treatments for PC involve surgery and chemotherapy. Nevertheless, a mere 15 to 20% of patients are eligible for surgery, and post-surgical relapse is highly probable. Moreover, PC tissue exhibits low blood vessel density and a fibrotic barrier, impeding the penetration of chemotherapy drugs and leading to drug resistance [64,65]. Although targeted therapies are available for certain PC subtypes associated with specific genetic mutations, their effectiveness is constrained by high costs, drug resistance, and the unique tissue characteristics of the pancreas [66].

Challenges in the diagnosis and treatment of PC emphasize the urgent need for innovative assisted diagnostic technologies. AI has the potential to reduce the burden on doctors and patients as it can automatically and accurately analyze medical images of PCs in a short time, which will bring tremendous time and economic benefits.

## 4. Public Data Sources

Public data sources of pancreatic medical images are essential for medical researchers, data scientists, and healthcare professionals. These datasets offer a rich source of visual information related to pancreatic tissues and cancerous cells. They facilitate research in medical imaging, machine learning, deep learning, and data science. Access to these datasets advances AI systems for analyzing pancreatic images. Detailed information on the currently publicized medical images of the pancreas used for research is provided below.

### 4.1. NIH (National Institutes of Health) [67]

This dataset comprises 82 abdominal CT scans of the pancreas. The scans have been enhanced with contrast for better visualization and have a resolution of 512 × 512 pixels. It includes 53 male and 27 female subjects, aged between 18 and 76 years. Among the subjects, there are 17 healthy kidney donors and additional patients without pancreatic lesions. To ensure accurate labeling, a medical student manually labeled each slice of CT scans, under the supervision of an experienced radiologist. This dataset offers an opportunity to investigate the pancreas across different age groups, genders, and health conditions.

### 4.2. AbdomenCT-1K [68]

This data set proposed by Ma et al. [68] contains more than 1000 CT images from 12 medical centers for large-scale studies of the segmentation of the liver, kidney, spleen, and pancreas to improve the generalizability of state-of-the-art models. They also establish benchmarks for fully supervised, semi-supervised, weakly supervised, and continuous learning segmentation and develop corresponding models for each benchmark.

### 4.3. BTCV (Beyond the Cranial Vault Multi-Organ Segmentation Challenge) [69]

This dataset comprises 50 abdominal CE-CT scans (30 for training and 20 for testing) obtained from the Vanderbilt University Medical Center, with 13 organs (including the pancreas) labeled in each scan, to perform a 13-class segmentation task. Each scan is made up of 80 to 225 slices, each with a resolution of 512 × 512 pixels. The images were manually labeled by the evaluator and precision was checked by a radiologist.

### 4.4. WORD (Whole Abdominal Organ Dataset) [70]

This dataset comprises 150 abdominal CT scans (100 for training, 20 for validation, and 30 for testing) of 30,495 slices and is the first whole abdominal organ dataset. Each scan is annotated with fine pixel-level annotations for 16 organs (including the pancreas) and sparse graffiti-based annotations, a weakly supervised method that reduces labeling costs.

### 4.5. MSD (Medical Segmentation Decathlon) [71]

This dataset comprises 420 portal venous phase CT scans of PC patients who underwent resection at Memorial Sloan Kettering Cancer Center in New York. The masses include IPMNs, pNET, and PDAC. An abdominal radiologist manually segmented the pancreatic parenchyma and pancreatic mass, including cysts or tumors, on each slice using the Scout application.

**Figure 3:**
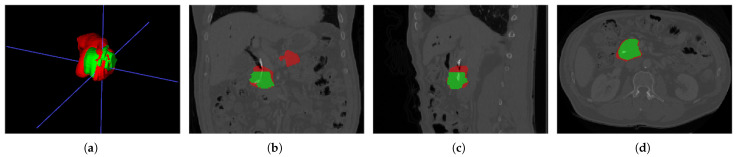
MSD sample data pancreas_004.nii.gz: (**a**) 3D visualization of pancreas and PC, (**b**) main view, (**c**) left view, and (**d**) top view.

As shown in Figure 3, ITK-SNAP [72] was used to visualize CT images, where the red labels represent healthy pancreatic tissues and the green parts represent PC tissues. The task of this dataset is to subdivide the pancreas and the PC, the latter being relatively challenging.

### 4.6. Dataset of Manually Segmented Pancreatic Cystic Lesions in CT Images [73]

The dataset contains 221 CT images with 543 pancreatic cystic lesions. Pancreatic cyst and main pancreatic duct are manually labeled in each CT image. In addition, it contains two nnUNet [74] models, one for segmentation of the pancreas, and one for segmentation of the cysts and main pancreatic duct.

### 4.7. TCGA (The Cancer Genome Atlas) [75]

The TCGA is a public-funded project to discover the causes of cancers. It involves multiple cooperating centers that collect, process, and analyze cancer samples. TCGA provides various types of cancer-related data, including diagnostic information, tissue, samples, and radiological images. Radiological images, such as those available in the NIH [67], can be found in the Cancer Imaging Archive (TCIA). Additionally, pancreatic pathological images are accessible through the GDC portal. These resources contribute to the comprehensive collection of data in the TCGA project, aiding researchers in understanding cancer and its characteristics.

### 4.8. SEER (Surveillance, Epidemiology, and End Results Program) [41]

The SEER program is designed to collect cancer statistics for research to mitigate the effects of cancer. A pancreatic tissue microarray (TMA) containing tumor tissue slides from 161 cases diagnosed between 1983 and 2000 has been established. Of these cases, 154 are PDAC and 7 are pNET. The primary objective of TMA is to explore the potential prognostic significance of PC tissue slides.

### 4.9. The PANORAMA Challenge (Pancreatic Cancer Diagnosis: Radiologists Meet AI) [76]

This dataset comprises CE-CT scans, including those from PDAC and non-PDAC patients. The non-PDAC group includes both individuals with healthy pancreas and those with non-PDAC pancreatic lesions. A separate test set of 400 scans has been prepared. This challenge aims to assess the clinical feasibility of modern pancreas-AI solutions for PDAC detection and diagnosis using CE-CT imaging.

### 4.10. LEPset [77]

**Figure 4:**
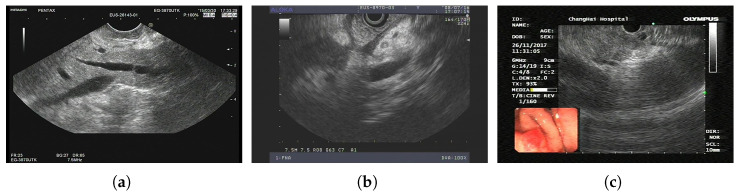
LEPset sample data: (**a**) labeled non-PC, (**b**) labeled PC, and (**c**) unlabeled image.

The dataset is based on EUS and consists of 420 patients and 3500 images. Its task is to classify PCs and non-PCs. Experienced physicians annotated these 3500 images with category labels. In addition, there are 8000 unlabeled images for pretraining. Sample images from LEPset are shown in Figure 4, with images labeled as PC or non-PC and unlabeled data.

### 4.11. PAIP 2023 (Tumor Cellularity Prediction in Pancreatic Cancer) [78]

**Figure 5:**
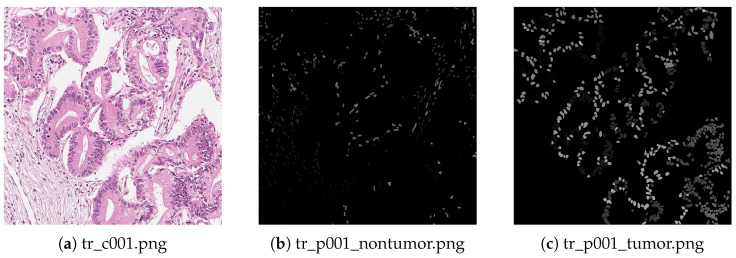
PAIP sample data: (**a**) a pathological image of PC, (**b**) nontumor cell nucleus mask, (**c**) tumor cell nucleus mask (The masks were processed to be visible).

This dataset comprises 80 pancreatic pathological images (50 for training, 10 for validation, 20 for testing) for tumor cell segmentation, with a resolution of 1024 × 1024. They utilize tumor cellularity (TC) as a metric between 0 and 100 to measure the remaining tumor burden in organs. The task of this dataset is to segment the tumor cell nucleus and calculate the TC. Figure 5 shows sample images from the training set, where each pathological image corresponds to two masks, representing the TC of the tumor cell nucleus and the non-tumor cell nucleus, respectively.

### 4.12. Dataset Related to Article of Grizzi et al. [79]

The dataset contains 7 patients with PDAC, 6 with chronic pancreatitis, and 5 with normal pancreas. Each category includes 10 pathological images for each case at 20× objective. The objective of the dataset is to accurately quantify the amount of pancreatic collagenic extra-cellular matrix, its spatial distribution patterns, and degradation processes by computer-assisted methods.

## 5. AI Tasks, Models, and Evaluation Metrics

**Figure 6:**
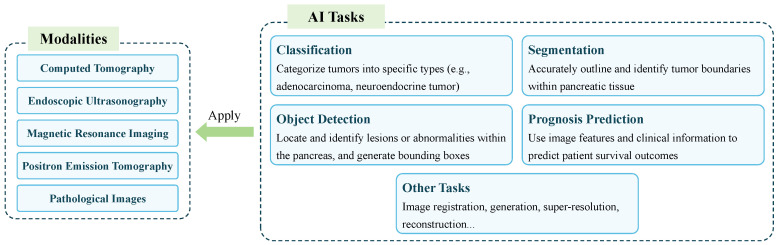
Summary of AI tasks on different medical imaging modalities.

In pancreatic image analysis, researchers mainly focused on four popular AI tasks: segmentation, classification, object detection, and prognosis prediction. Figure 6 summarizes the main AI task applications in different image modalities. To comprehensively and objectively assess the performance of an AI task, appropriate metrics must be used. This section introduces the metrics used in this review, which are widely recognized and used widely. By utilizing these metrics, researchers can assess the effectiveness of various tasks in a standardized and comparable manner.

**Figure 7:**
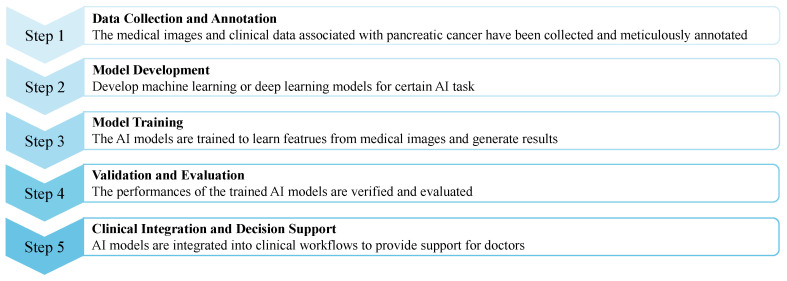
Flowchart of AI application in pancreatic images analysis.

The workflow of AI-enabled automated PC analysis is shown in Figure 7. The medical images and clinical data (if available) are first collected and annotated; then, the data will be pre-processed as the input of AI models for a certain task. Next, the AI models are trained to learn the features and generate corresponding results. Finally, AI models can be applied to support clinical workflows after they have been assessed as reliable.

### 5.1. Classification

#### 5.1.1. Introduction to Classification

Image classification is a well-founded task in computer vision that aims to assign labels or categories to the input image as a whole based on its content. This task forms the basis for various applications. The classification of PCs is categorizing medical images into distinct types, including PC and non-PC cases or different subtypes of PC within an image. Classification focuses on recognizing overall patterns and characteristics, rather than providing precise tumor boundaries. Specifically, some basic methods of classification using AI models are shown below:

**Feature extraction + machine learning** Manually extracted features are often interpretable, helping to understand the physical or biological characteristics behind the data, allowing for better control over the model’s input, and reducing noise and unnecessary information. In medical image classification, the process of feature extraction typically involves several common methods and follows a systematic workflow. Firstly, regions of interest (ROIs) are delineated within the images, focusing on areas relevant to the diagnostic task. Subsequently, various feature extraction techniques are applied to these ROIs including shape features, which encompass parameters like height, width, perimeter, area, and others to describe geometric properties. Texture features are extracted using methods such as gray-level co-occurrence matrix (GLCM), gray-level run length matrix (GLRLM), gray-level gradient co-occurrence matrix (GLGCM), and gray-level distribution statistics (GLDS). Additionally, the wavelet transform can also be utilized to capture multiscale texture information. Following feature extraction, a feature selection step is often performed to reduce dimensionality and remove irrelevant or redundant features. Finally, normalization techniques may be applied to ensure that features are on a comparable scale.

**Figure 8:**
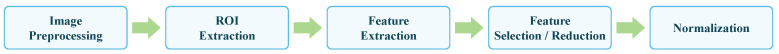
Basic workflow of feature engineering in traditional machine learning based image classification.

The feature extraction workflow can be seen in Figure 8, and then the normalized data will be fed into machine learning models. Commonly used machine learning models include supervised learning and unsupervised learning. In supervised learning, common classification models include logistic regression, decision trees, k-nearest neighbor (KNN), support vector machines (SVMs), random forests (RFs), naïve Bayes, and so on. In unsupervised learning, k-means clustering is usually performed on unlabeled data.

Steps of ROI extraction can be performed manually or by segmentation using deep learning algorithms (the segmentation task will be described in a later section). Segmentation can be performed as a separate feature extraction step or included in an end-to-end deep learning workflow.

**Deep features + machine learning** Using deep features and machine learning for image classification combines the powerful feature extraction capabilities of pretrained CNNs with the robust performance of traditional machine learning algorithms. In this approach, high-level features are extracted by pretrained CNNs from images, which are input to machine learning algorithms. The primary advantage is the combination of deep learning’s feature extraction strength without training from scratch and traditional algorithms’ efficiency and flexibility. However, a key disadvantage is that pretrained models might not always capture the specific characteristics of the target image dataset, potentially leading to suboptimal feature representation and requiring fine-tuning or additional domain-specific training.

**End-to-end deep learning** Using deep learning models like CNNs or Transformers directly for image classification can automatically learn features from raw data without time-consuming manual feature extraction. The evolution of image classification models has witnessed a shift towards increasingly sophisticated architectures and techniques. Initially, pioneering models like AlexNet [80] and VGG [81] emphasized the importance of deeper networks for capturing intricate image features. Subsequently, ResNet [82] introduced residual connections, enabling the training of even deeper networks while mitigating the vanishing gradient problem. Furthermore, attention mechanisms have emerged as a pivotal component in image classification, as evidenced by the great success of transformer-based architectures like vision transformer (ViT) [83] and swin transformer [84]. These models leverage self-attention mechanisms to capture global dependencies and contextual information, allowing for more effective feature representation and classification.

**Feature extraction + deep learning** Some methods, while employing deep learning techniques, do not directly input images but instead undergo a feature extraction process. This approach allows for leveraging both the power of neural networks’ learning ability and the explainability of manual feature extraction. However, in this case, neural networks only act as classifiers rather than feature extractors. It is unnecessary to use a very deep neural network that may involve additional computational costs. Most times, a shallow one is enough.

**Deep learning + machine learning** There are also works combining traditional machine learning methods with deep learning techniques. Traditional machine learning plays a role in integrating multiple deep learning models, reducing the risk of overfitting and capturing a broader range of patterns present in the data. Additionally, ensemble methods can compensate for the weaknesses of individual models, leading to improved overall performance.

#### 5.1.2. Evaluation Metrics for Classification

**Accuracy** Accuracy measures the proportion of correctly classified instances out of the total instances. It quantifies the overall correctness of the classifier’s predictions. The Accuracy ranges from 0 to 1, where 1 indicates the predictions are all correct, while 0 indicates the model does not make any correct predictions. Accuracy is calculated by

(1) $$\mathrm{Accuracy}=\frac{\mathrm{TP}+\mathrm{TN}}{\mathrm{TP}+\mathrm{TN}+\mathrm{FP}+\mathrm{FN}},$$

where true negatives (TNs) represent instances correctly predicted as negative, true positives (TPs) represent the number of instances correctly predicted as positive, false positives (FPs) represent the number of instances incorrectly predicted as positive when they are negative. False negatives (FNs) represent the number of instances incorrectly predicted as negative when they are positive.

**Specificity** Specificity is the probability of a negative test result and refers to the ability of the test to correctly reject a healthy patient without disease. A positive result on a high specificity test close to 1 can be used to diagnose a disease, as the test rarely gives a positive result in a healthy patient [85]. Specificity is calculated by

(2) $$\mathrm{Specificity}=\frac{\mathrm{TN}}{\mathrm{TN}+\mathrm{FP}}.$$

**Precision** Precision is the proportion of relevant instances among the retrieved instances. Higher precision means that the algorithm returns more relevant results than irrelevant ones. Precision is calculated by dividing the number of true positives by the total number of elements labeled as belonging to the positive class:

(3) $$\mathrm{Precision}=\frac{\mathrm{TP}}{\mathrm{TP}+\mathrm{FP}}.$$

**Recall** Recall is the probability of a positive test result and refers to the ability of the test to correctly detect a sick patient. A Recall close to 1 can be used to rule out a disease because it rarely misdiagnoses someone with the disease [85]. Recall is calculated by

(4) $$\mathrm{Recall}=\frac{\mathrm{TP}}{\mathrm{TP}+\mathrm{FN}}.$$

**Area Under Curve (AUC)** AUC is based on receiver operating characteristic (ROC) curves, which are plots of true-positive rate (TPR) versus false-positive rate (FPR) for each threshold setting. The AUC calculates the area under the ROC curve and summarizes the sensitivity and specificity, but does not provide information on the precision and negative predictive value. AUC is calculated by

(5) $$\mathrm{AUC}=\int_{0}^{1}\mathrm{TPR}\left(f\right),d\mathrm{FPR}\left(f\right),$$

where TPR(f) represents the TPR at a specific threshold *f*, and dFPR(f) represents the FPR at the same threshold *f*.

**F1-Score** The F1-score is calculated from the Precision and Recall of the test and represents them symmetrically. The highest possible value for the F1-score is 1.0, which indicates perfect Precision and Recall; the lowest value is 0 if Precision and Recall are 0. The F1-score is calculated by

(6) $$F1\text{-}\mathrm{score}=2\times \frac{\mathrm{Precision}\times \mathrm{Recall}}{\mathrm{Precision}+\mathrm{Recall}}.$$

### 5.2. Segmentation

#### 5.2.1. Introduction to Segmentation

Image segmentation refers to the precise outlining of the boundaries of the pancreatic organ or PC. With accurate pancreas and PC boundaries, doctors can get the size, shape, location, and other features of the PC for quick diagnosis and treatment planning. Instance segmentation and semantic segmentation are the two basic branches of the segmentation task. Semantic segmentation categorizes each pixel in an image into a predefined class without distinguishing different instances of the same class. Further, instance segmentation not only categorizes pixels but also distinguishes between individual object instances of the same class, assigning unique labels to each instance. Several popular segmentation topics and methods are as follows:

**Superpixel extraction** Early-stage pancreas and PC segmentation before the proposal of FCN relies on superpixel extraction. Superpixel extraction in medical image segmentation involves grouping pixels with similar characteristics to form cohesive regions. Algorithms like simple linear iterative clustering (SLIC) or quickshift partition the image into superpixels based on color, intensity, or texture similarities, followed by feature extraction and segmentation to assign labels to each region.

**FCN/UNet-based methods for 2D segmentation** Fully convolutional neural networks (FCNs) [86] revolutionized segmentation by enabling end-to-end learning, allowing pixel-wise predictions directly from input images and offering greater flexibility, efficiency, and performance in image semantic segmentation tasks compared to superpixel selection. Based on this architecture, UNet [87] stands out for its U-shaped design, efficiently capturing both high-level context and precise localization information. A lot of UNet’s variants like Attention UNet [88], UNet++ [89], ResUNet++ [90], Channel UNet [91], UNet3+ [92] and so on are proposed aiming at refining segmentation accuracy and addressing specific challenges.

**FCN/UNet-based methods for 3D segmentation** Three-dimensional (3D) segmentation in medical imaging offers enhanced accuracy and comprehensive visualization by considering the entire volume of the image, facilitating precise treatment planning, quantitative analysis, and time efficiency in clinical practice. Unlike its 2D counterpart, which processes images as two-dimensional matrices, 3D convolution considers the depth, height, and width of the input volume, using three-dimensional kernels to capture spatial dependencies along all three axes. This extension facilitates the modeling of complex volumetric structures and temporal dynamics, making 3D convolution well-suited for tasks involving volumetric medical imaging, video processing, and any application where understanding three-dimensional spatial relationships is essential for accurate analysis. Models like 3D U-Net [93], S3D-UNet [94], and V-Net [95] are advanced 3D CNN architectures designed to segment volumetric medical image.

**Figure 9:**
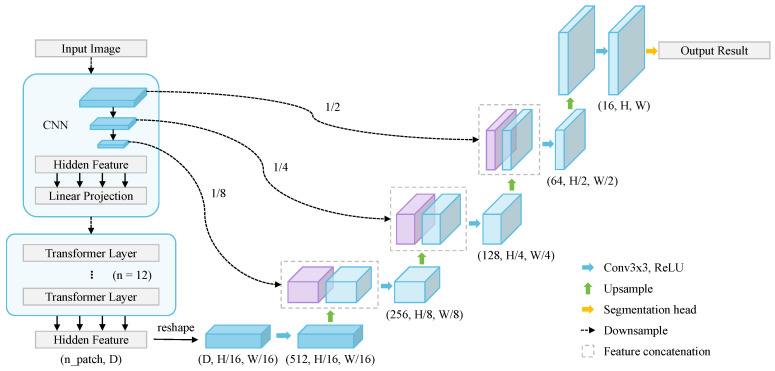
TransUNet architecture.

**Transformers for 2D medical image segmentation** While FCN/UNet-based approaches have been highly successful, their convolutional layers suffer from flaws in accessing global and remote long-range semantic information. Thus, more and more attention is paid to the success of ViT in visual tasks. Transformers can offer significant advantages in 2D medical segmentation for capturing global context information, handling variable input sizes, leveraging attention mechanisms for focusing on relevant features, and utilizing pretrained models for transfer learning, ultimately improving segmentation accuracy and performance in medical imaging tasks. Transformer-based UNet variants including pure Transformer models and hybrid models of Transformer and CNNs. Typical models like TransUNet [96], Swin-UNet [97], Transformer-UNet [98], and TransAttUNet [99] show great segmentation performance on a series of medical image segmentation tasks. Figure 9 shows the structure of TransUNet, a representative model that merits both Transformers and UNet. This framework utilizes a CNN-Transformer hybrid encoder to generate the feature map, and then a cascaded upsampler decodes the hidden feature and outputs the segmentation results.

**Figure 10:**
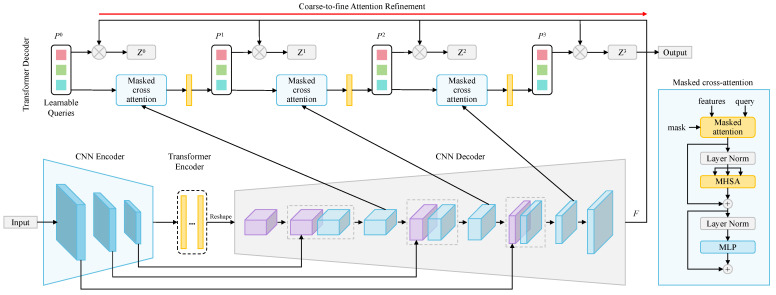
3D TransUNet architecture.

**Transformers for 3D medical image segmentation** The volume medical image segmentation task can also be redesigned as sequence-to-sequence prediction. The transformer operates on a sequence of input embeddings of a 3D input volume $x\in R^{H\times W\times D\times C}$ with resolution (H,W,D) and *C* input channels by dividing it into flattened uniform nonoverlapping patches $x_{v}\in R^{N\times \left(P^{3}.C\right)}$, where (P,P,P) denotes the resolution of each patch and $N=\left(H\times W\times D\right)/P^{3}$ is the length of the sequence. Figure 10 shows the structure of 3D TransUNet [100], an extension of TransUNet for 3D image segmentation.

**Graph-based methods for medical image segmentation** Graph-based methods utilize the concept of graph theory to represent the image as a graph, where pixels or voxels are nodes, and their relationships are represented by edges. Graph neural networks like graph convolution networks (GCNs) [101], graph attention networks (GATs) [102], and graph isomorphism networks (GINs) [103] can be used to process such graph representation.

**Instance Segmentation** Instance segmentation algorithms can be divided into three main branches: two-stage methods like mask R-CNN [104] and cascade mask R-CNN [105] employ a two-step process involving region proposal generation followed by mask refinement; one-stage methods like SOLO [106] and SOLOv2 [107] directly predict object categories and segmentation masks without separate proposal steps, offering efficiency at the cost of some accuracy; emerging query-based approaches, such as QueryInst [108], formulate instance segmentation as a query-driven interaction problem.

#### 5.2.2. Evaluation Metrics for Segmentation

**Dice Similarity Coefficient (DSC)** DSC indicates the ratio of overlapping pixels between the prediction and ground truth masks to the total number of pixels in the two masks. The coefficient ranges from 0 to 1, where 1 indicates a perfect match between the predicted and ground truth masks, and 0 indicates no overlap. DSC is calculated by

(7) $$\mathrm{DSC}=\frac{2\times |A\cap B|}{\left|A|+|B\right|},$$

where *A* represents the predicted segmentation mask or set of pixels, and *B* represents the ground truth segmentation mask or set of pixels.

**Intersection over Union** IoU, also known as the Jaccard Index, measures the overlap between a predicted region and a ground truth region. The IoU ranges from 0 to 1, where 1 indicates a perfect overlap between the masks, and 0 indicates no intersection. IoU is calculated by

(8) $$\mathrm{IoU}=\frac{\left|A\cap B\right|}{\left|A\cup B\right|}.$$

Based on IoU, mean IoU (mIoU) is calculated as the average of the IoUs for each pixel class:

(9) $$\mathrm{mIoU}=\frac{1}{N}\sum_{i=1}^{N}{\mathrm{IoU}}_{i},$$

where *N* is the number of pixel classes. Instance segmentation tasks typically use mIoU to evaluate performance, averaging segmentation results across multiple categories.

**Other Metrics** In addition to DSC and IoU, there are also distance-based metrics such as Hausdorff distance (HD) [109] and normalized surface distance (NSD) [110]. These metrics consider the distances between two sets of shapes that quantify the difference between the segmentation result and the true label. The larger the distance, the greater the difference between the two shapes, and the worse the performance of the models.

The HD is calculated by

(10) $$\begin{array}{c} H(A,B)=\max(h(A,B),h(B,A)),\\ h\left(A,B\right)=\max_{a\in A}\min_{b\in B}∥a-b∥, \end{array}$$

where H(A,B) is the HD between A and B, h(A,B) is the directed HD, and ∥·∥ is some underlying norm on the points of *A* and *B*.

The NSD is calculated by

(11) $$\mathrm{NSD}=\frac{|S_{i}\cap B_{j}^{\left(\tau \right)}\left|+\right|S_{j}\cap B_{i}^{\left(\tau \right)}|}{|S_{i}\left|+\right|S_{j}|},$$

where τ is the tolerance, $S_{i}$ and $S_{j}$ are surfaces, $B_{i}$ and $B_{j}$ are border regions, *i* is the prediction, and *j* is the reference.

Moreover, Accuracy, Specificity, Recall, Precision, AUC, and F1-score can also be used to evaluate segmentation tasks, as discussed in Section 5.1. Unlike classification, these metrics evaluate the performance of the segmentation models in terms of their prediction at each pixel point.

### 5.3. Object Detection

#### 5.3.1. Introduction to Object Detection

Object detection refers to the classification and localization of objects in an image, which involves assigning labels to each detected object and providing a bounding box around the object. By accurately detecting and localizing objects in an image, object detection algorithms enable machines to effectively understand and interact with visual information.

**2D Object detection** Two-dimensional (2D) object detection can be categorized into two main approaches: single-stage and two-stage methods. Single-stage methods, such as and SSD [111] and the YOLO (you only look once) series [112,113,114,115,116,117,118,119], perform object detection in a single step. They directly predict object bounding boxes and class probabilities from the entire image using a unified network architecture. While these methods are faster, they may sacrifice some accuracy compared to two-stage methods. Two-stage methods, like R-CNN [120], fast-RCNN [121], and faster R-CNN [104], divide the object detection process into region proposal generation stage and object classification stage. In the first stage, region proposal networks generate potential object bounding boxes, which are then refined and classified in the second stage. These methods typically achieve higher accuracy but require more computational resources. Transformer-based methods like DETR [122] can capture global context and long-range dependencies in images more effectively. This approach enables accurate object detection by attending to relevant image regions and preserving spatial information through positional encodings.

**3D object detection** Three-dimensional (3D) object detection is also beneficial for clinical practice. Volume of interest (VOI) extraction is a crucial preprocessing step that enables tasks like organ segmentation and tumor classification by localizing relevant structures, reducing computational burden, and enhancing accuracy [123]. The main methods for 3D bounding box detection include generating from 2D box detection [124], generating from coarse segmentation [125], reinforcement learning [126], 3D detection models like using 3D region proposal network [127] and so on.

#### 5.3.2. Evaluation Metrics for Object Detection

**IoU** In addition to segmentation, IoU could also be used to assess the performance of object detection. In formula

(12) $$\mathrm{IoU}=\frac{\left|A\cap B\right|}{\left|A\cup B\right|},$$

*A* and *B* represent the predicted and ground truth bounding boxes, respectively.

**mAP** mAP is the mean value of Average Precision (AP). The AP involves computing the Precision and Recall values for each class and then integrating Precision to Recall. The integration is performed using the area under the Precision–Recall curve. AP is calculated by

(13) $$\mathrm{AP}=\frac{1}{n}\sum_{k=1}^{n}\left(P\left(k\right)\times \mathrm{rel}\left(k\right)\right),$$

where *n* represents the total number of relevant items in the retrieved set, P(k) represents the Precision at cut-off *k*, rel(k) is an indicator function equaling 1 if the item at rank(k) is relevant, and 0 otherwise. mAP is calculated by

(14) $$\mathrm{mAP}=\frac{1}{C}\sum_{i=1}^{C}{\mathrm{AP}}_{i},$$

where *C* represents the total number of object classes, ${\mathrm{AP}}_{i}$ represents the AP for class *i*. mAP@0.5 measures the mAP when the intersection over union (IoU) threshold for considering a detection as a true positive is 0.5. mAP@0.5:0.95 measures the mAP averaged over different IoU thresholds ranging from 0.5 to 0.95, typically in increments of 0.05.

**Other Metrics** If the IoU is greater than a set threshold (e.g., 0.5), the predicted bounding box can be treated as a correct detection. The metrics mentioned by Section 5.1 (accuracy, specificity, recall, precision, AUC, and F1-score) can be obtained from the number of correctly predicted bounding boxes versus the number of incorrectly predicted bounding boxes. IoU greater than the threshold is classified as TP, IoU less than the threshold as FP, and IoU of 0 as FN.

### 5.4. Prognosis Prediction

#### 5.4.1. Introduction to Prognosis Prediction

The prognosis prediction for PC patients integrates medical images and clinical data to forecast the survival period of individuals. This predictive capability assists doctors in making informed prognosis decisions for their patients.

Prognosis prediction is to predict outcomes for PC patients after undergoing surgery, such as predicting whether or not the patient will survive after some time, or for overall survival (OS) time. The predicted results will usually be compared with the real results for consistency, thus evaluating the model performance. Basic methods for prediction of prognosis typically include machine learning algorithms such as least absolute shrinkage and selection operator (LASSO) regression, SVM, or more advanced techniques such as random forests (RF) or gradient enhancement. These methods leverage the relationship between input features, such as medical imaging characteristics and clinical variables, and the target variable, such as survival time, to learn predictive models to accurately forecast patient outcomes. Features like tumor size, shape, and texture are traditionally manually extracted from images to inform prognosis models. With the advent of deep learning, automatic feature extraction has gained traction. CNNs autonomously learn discriminative features directly from images, eliminating the need for manual feature engineering.

#### 5.4.2. Evaluation Metrics for Prognosis Prediction

**Concordance Index** The C-index is used to assess the predictive model accuracy in survival analysis. It measures how well a model ranks the relative order of survival times for different individuals. The C-index ranges from 0 to 1, with higher values indicating better predictive accuracy. A C-index of 1 indicates perfect concordance, where the model consistently ranks survival times correctly. A C-index of 0.5 represents a random prediction, indicating that the model’s predictions are not informative. C-index is calculated by

(15) $$C\text{-}\mathrm{index}=\frac{N_{C}}{N_{T}},$$

where $N_{C}$ represents the number of concordant pairs, and $N_{T}$ represents the number of all evaluable pairs. Concordant pairs are pairs of individuals where the predicted survival times have the same relative order as the actual survival times. Comparable pairs are pairs where a meaningful comparison can be made, excluding tied or censored survival times.

**Other Metrics** Prognosis prediction also includes predicting whether a patient will die and whether PC will develop metastasis within a period after treatment. In this case, the previously mentioned metrics Accuracy, Specificity, Precision, Recall, AUC, and F1-score could evaluate the AI models on prognosis prediction task as well.

### 5.5. Other Tasks

In addition to the mentioned tasks, there are several other common AI tasks in medical image analysis that could potentially aid in the diagnosis and treatment of PC. These include registration as well as various low-level visual tasks.

Image registration involves aligning multiple images from different sources or times, aiding in tracking disease progression or integrating data from different imaging modalities. Image generation encompasses techniques for creating new images based on existing ones, such as generating synthetic images to augment training data or simulating different imaging scenarios for educational purposes. Super-resolution techniques enhance image resolution, enabling the detection of finer details in pancreatic imaging and aiding in the identification of smaller lesions or abnormalities. Denoising methods remove noise from images, improving clarity, and facilitating the identification of relevant features in pancreatic images, especially against a noisy background. Reconstruction involves creating complete images from partial or incomplete data, such as reconstructing 3D images from 2D scans. The Medical Visual Question Answering (MedVQA) task combines computer vision and natural language processing (NLP) by analyzing input medical images and related questions and outputting answers to inform medical diagnosis and treatment. The above-mentioned tasks utilize a variety of AIs that can help improve the quality of imaging data and the accuracy of automated analysis, helping physicians diagnose and treat PCs more effectively.

## 6. Computed Tomography (CT)

### 6.1. Introduction to CT

CT, also known as computed axial tomography (CAT), is a noninvasive imaging technique that rapidly produces three-dimensional imaging of the inside of the body. It is the most widely used radiologic imaging method and has become a standard. Compared to conventional radiography, CT offers higher contrast. The advent of CT revolutionized the field of medical imaging, becoming an indispensable tool for diagnosis and treatment [128]. CE-CT uses iodinated contrast agents to increase the visibility of blood vessels, distinguishing them from their surroundings. This approach increases clarity and provides more detail to better analyze anatomy and potential abnormalities. However, iodinated contrast agents also have side effects, such as causing nephropathy [129].

### 6.2. Classification

**Feature extraction + machine learning** Li et al. [130] used six methods for feature extraction, as shown in Table 3, and the LASSO algorithm for feature selection, and then applied the EL-SVM learner to classify normal pancreas, early-stage (stage I and stage II), stage III, and stage IV of PC. Chen et al. [131] trained an XGBoost [132] model to classify patches as cancerous or noncancerous. Patients were classified as either PDAC or non-PDAC based on the proportion of patches classified as cancerous. Mukherjee et al. [133] conducted feature extraction, normalization, and reduction, and trained four independent ML classifiers known as KNN, SVM, RF, and XGBoost to recognize PDAC at the prediagnostic stage, which achieved high accuracy.

**End-to-end deep learning** Liu et al. [11] used the VGG model to differentiate PC tissue from noncancerous pancreatic tissue. Xia et al. [134] proposed a deep classification model that combined UNet with Anatomy-aware Hybrid Transformers using a single-phase noncontrast CT to facilitate more accurate, safe, and low-cost screening for distinguishing between PDAC, other abnormalities, and normal pancreas. Cao et al. [135] introduced PC detection with artificial intelligence (PANDA) method to detect and classify pancreatic lesions based on the lesion segmentation results of nnUNet. CNNs with a classification head were used to classify PDAC, pNET, SPT, IPMN, MCN, chronic pancreatitis, SCN, etc. Segmentation and classification models are included in an end-to-end scheme.

**Feature extraction + deep learning** Vaiyapuri et al. [136] proposed an IDLDMS-PTC technique to examine the CT images for the existence of pancreatic tumors. The proposed technique comprises several sub-processes: GF-based pre-processing, EPO-MLT-based segmentation, MobileNet-based feature extraction, AE-based classification, and MLO-based parameter optimization. Huy et al. [137] used Densenet to distinguish cancerous tumors from benign tumors in CT pancreatic images.

**Deep learning + machine learning** To classify pancreatic SCNs and MCNs, Yang et al. [138] applied a multichannel-multiclassifier-RF-ResNet (DNN-MMRF-ResNet). SVM, KNN, and Bayes classifiers were used after the residual block, and then the final classification was finished by an RF classifier. Bakasa et al. [139] utilized Inception V3, VGG16, and ResNet34 as weak learners in a stacking ensemble, where their first-level predictions formed the input for XGBoost that performed the final pancreas cancer classification.

Table 4 shows the comparison of AI models in CT pancreatic images for the classification task.

### 6.3. Segmentation

**Superpixel extraction** Roth et al. [140] extracted superpixels from the abdominal region are extracted via SLIC. Initial probability response maps are generated using a two-level cascade of RF classifiers, retaining superpixels with probabilities above 0.5, followed by CNN sampling bounding boxes at various scales and nonrigid deformations for refined pancreas region identification. Roth et al. [67] introduced a probabilistic bottom-up approach to segment the pancreas in abdominal CT scans, employing multilevel deep CNNs. Various ConvNets variations are evaluated for hierarchical classification on image patches and regions (superpixels), with post-processing using structured predictions.

**FCN/UNet-based methods for 2D segmentation** Heinrich and Oktay [141] developed BRIEFnet, which utilized binary sparse convolutions in CNNs to reduce memory cost and improve segmentation performance. Zhou et al. [142] utilized pretrained FCN-8s incorporated with deeply-supervised nets (DSN) [143] to develop a coarse-to-fine segmentation algorithm. The model obtained a reasonable segmentation of pancreatic cysts. Lu et al. [144] proposed a Ringed Residual U-Net using the ring residual module as well as the attention mechanism. Boers et al. [145] implemented the interactive method iFCN and introduced iUNet, an interactive version of the U-net method, which is fully trained for optimal initial segmentation and additionally fine-tuned on user-generated scribbles in interactive mode. Jiang et al. [146] proposed DLU-Net with deformable convolution modules to strengthen the ability to model the target edge, and the Bi-Directional Convolutional Long-Short Term Memory (BConvLSTM) was utilized to merge the features of different scales. Li et al. [147] used the skip network, residual network, and multiscale residual network strategies to efficiently address over- and under-segmentation issues through cross-domain connections and multiscale convolution operations, enhancing accuracy in pancreas shape learning. Li et al. [148] proposed a Window Attention Upsample (WAU) for upsampling, consisting of an Attention Decoder (AD) and a bilinear upsample. A window attention scheme is used to reduce computation by restricting computation in local windows instead of the global range. Paithane and Kakarwal [149] introduced a 12-layer LMNS-net with 4 convolution layers, where a lightweight multiscale block dropped the unused information. Juwita et al. [150] proposed M3BUNet, which fused MobileNet and UNet and was equipped with Mean-Max attention. In addition, they utilized a coarse-to-fine segmentation process to improve performance.

Some methods first locate the organ of interest, such as the pancreas, and then identify any abnormalities or lesions within it. This two-step process involves initially segmenting the organ from surrounding structures and then focusing on regions of interest within the organ for further analysis. Zhou et al. [151] proposed DBFE-Net with two branches. DB-Net is used to extract semantic and fine-grained features for pancreas segmentation with a coarse-to-fine strategy, and then FE-Net is used to extract fine-grained features with higher contrast for tumor segmentation in the pancreas region.

Some works focus on utilizing spiral transformation to map 3D images onto 2D planes while preserving spatial relationships, facilitating effective 3D contextual information utilization in a 2D model. Chen et al. [152] applied spiral transformation for data augmentation and incorporated a transformation-weight-corrected module based on Res-UNet [153]. This design addressed small sample size issues and ensured uniform 3D segmentation and rebuilding constraints, overcoming nonunique 3D results from uniform sampling.

**FCN/UNet-based methods for 3D segmentation** Roth et al. [154] investigated the 3D U-Net of two types of pancreas segmentation, one with concatenation and one with summation skip connections. Chen et al. [155] introduced a new bias-dice loss function for improved efficiency in 3D coarse segmentation, utilizes a dimension adaptation module (DAM) to incorporate 3D information into 2D networks, and proposes a fusion decision module and parallel training strategy to integrate multisource feature cues from sub-networks for final predictions. Zhao et al. [156] proposed a two-stage framework that utilized a 3D UNet to provide candidate regions in the first stage, and another 3D UNet was trained to obtain the final results based on these candidates in the second stage. Zhang et al. [157] proposed a dynamic on-demand network (DoDNet) with a dynamic segmentation head, addressing the partially labeled issue in medical images and being applied to multiple tumors. They also proposed a large-scale partially labeled dataset MOTS for pretraining models. Zhang et al. [158] developed the scale-transferrable feature fusion module (STFFM) and prior propagation module (PPM) modules to simplify FCNs. STFFM utilized the scale-transferrable operation to learn rich fusion features, and PPM explored informative spatial priors by dynamically adapting the spatial priors to input and feature maps.

**nnUNet** Isensee et al. [74,159] introduced the no-new-Net (nnUNet), a robust and self-adapting framework based on 2D and 3D vanilla U-Nets without using various extension plugins (residual connections, Dense connections, and various attention mechanisms), which can automatically adapt architectures to image geometry. In addition, they defined steps for nnUNet: pre-processing, training, inference, and potential post-processing. Yao et al. [160] employed nnUNet for IPMN segmentation and achieved a better DSC than the previous studies. In recent years, nnUNet has achieved remarkable success and widespread application in medical image segmentation competitions, prompting a rethinking of the task. Effective preprocessing, post-processing, training, and inference strategies may be more important than complex network architectures.

**Transformers for 2D medical image segmentation** Sha et al. [98] proposed Transformer-Unet, which combined Transformer and UNet by replacing Transformer modules in raw images with feature maps in UNet. Huang et al. [161] introduced Medical Image Segmentation tranSFormer (MISSFormer), a hierarchical encoder-decoder network. They redesigned the feed-forward network with the Enhanced Transformer Block and used the Enhanced Transformer Context Bridge to extract long-range dependencies and local context of multiscale features. Chen et al. [96] proposed TransUNet that combined Transformers and UNet. The Transformer encoders tokenized image patches from the CNN feature map to obtain global contexts, and these encoded features were combined with high-resolution CNN feature maps for precise localization. Cao et al. [97] introduced Swin-UNet, a UNet-like pure Transformer that uses a hierarchical Swin Transformer encoder to extract context features and a symmetric decoder to restore spatial resolution. Dai et al. [162] put forward a two-stage Trans-Deformer network (TD-Net), with a 2D UNet for coarse segmentation and ViT for fine segmentation. In this framework, the multi-input module was designed to focus on high-frequency texture information, and the scale interactive fusion (SIF) module was designed to combine local and global features. Rahman et al. [163] proposed a medical image segmentation transformer (MIST) using convolutional attention mixing (CAM) to capture local contexts of pixels in multimodal dimensions.

**Transformers for 3D medical image segmentation** Zhou et al. [164] proposed not-another transFormer (nnFormer) that combined interleaved convolution and self-attention operations and utilized local and global volume-based self-attention mechanisms. Moreover, they replaced the traditional concatenation or summation in skip connections with skip attention in UNet-like architecture. Hatamizadeh et al. [165] put forward UNet Transformers (UNETR) utilizing a skip-connected transformer encoder to capture global multiscale information. Tang et al. [166] introduced Swin UNETR, a self-supervised framework, which utilized an encoder to extract features from multiple resolutions and was pretrained on 5050 public CT images. The model can also be applied to various proxy tasks after fine-tuning. Chen et al. [100] extended 2D TransUNet to 3D TransUNet, which tokenized image patches from a CNN feature map using a Transformer encoder and the Transformer decoder adaptively refined candidate regions by employing cross-attention between candidate proposals and U-Net features. Qu et al. [167] introduced a transformer-guided progressive fusion network (TGPFN), which supplemented long-range dependencies of convolutions by global representation captured by the transformer.

**Graph-based methods for medical image segmentation** Guo et al. [168] proposed a layered optimal graph image segmentation of multiple objects and surfaces (Deep LOGISMOS) method utilizing a UNet, trained on adjacent 2D patches centered at the tumor to provide contextual segmentation, refined by Gaussian Mixture Model (GMM) and morphological operations, followed by segmentation graph construction using UNet probability maps and a max-flow algorithm for globally optimal segmentation. Soberanis et al. [169] improved UNet based on uncertainty analysis and GCNs, which trained a GCN to solve a semi-supervised graph learning problem about the uncertainty levels of a particular input volume. Hu et al. [170] proposed a distance-based saliency-aware model (DSD-ASPP-Net), a coarse-to-fine framework that trained a Dense Atrous Spatial Pyramid Pooling (DenseASPP) model to learn location and probability map of the pancreas for coarse stage and saliency-aware modules for fine stage. Zhao et al. [171] introduced a holistic segmentation-mesh-classification network (SMCN) that combined geometry and location information and a graph-based residual convolutional network (Graph-ResNet) with nodes fused the information of the mesh model and feature vectors of the segmentation network. Liu et al. [172] developed a graph-enhanced pancreas segmentation network (GEPS-Net), which added a graph enhancement module to UNet to extract the spatial relationship information.

**Neural architecture search (NAS)** NAS optimizes segmentation models by automatically finding the best network structures for improved performance. It adjusts parameters like depth and width to suit specific tasks and datasets, overcoming limitations of manual design and enhancing model accuracy and efficiency. Zhu et al. [173] employed a NAS for volumetric medical image segmentation (V-NAS), which could choose 2D, 3D, or Pseudo-3D (P3D) convolutions at each layer automatically. He et al. [174] proposed the Differentiable Network Topology Search (DiNTS) scheme, including a topology-guaranteed discretization algorithm and a discretization-aware topology loss. Moreover, DiNTS could search 3D networks under different GPU memories and significantly reduce training time.

**Utilizing the power of large models** Large models, also known as foundation models, refer to deep learning models with extensive parameters and complex computational architectures, which can offer improved performance by capturing intricate patterns and relationships in data, enabling enhanced representation learning, flexibility across diverse domains, state-of-the-art results, and scalable handling of growing datasets and complex tasks. He et al. [175] found that SAM showed the lowest segmentation performance on the pancreas over 10 different organs (brain, chest, lung, liver, pancreas, prostate, bowel, skin, heart, and breast), and concluded that SAM is not as accurate as dataset specific deep learning algorithms in medical images. Therefore, the road to zero-shot segmentation for the pancreas and PC is still long. Mazurowski et al. [176]’s experimental datasets on SAM included MSD. Huang et al. [177] test the SAM on a built large medical dataset using different modes containing 18 modalities, 84 objects, 1050K 2D images, and 6033 masks. The sources of this collected dataset included AbdomenCT-1K related to the pancreas, promoting the research on zero-shot segmentation. Liu et al. [178,179] proposed the CLIP-Driven Universal Model based on Contrastive Language-Image Pretraining (CLIP) [180]. Using transfer learning on 3410 CT scans, they trained a universal model to capture anatomical relationships.

**Federated learning** Federated learning is one of the machine learning methods and allows model training on decentralized devices or servers, preserving local data samples while protecting user privacy and data security. Models can be learned from different data sources without sharing the original data, making it particularly suitable for applications in healthcare. Knolle et al. [181] proposed a shallow and U-Net-like framework MoNet based on repeated dilated convolutions with decreasing dilation rates. This framework reduces inference time and memory compared to UNet variants and is suitable for federated learning. Wang et al. [182] put forward the conditional distillation federated learning (ConDistFL) framework, which combined federated learning with knowledge distillation. This framework was trained on images of various organs and could extract knowledge of unlabeled tumors from labeled ones. Their study also increased the stability and reduced the training time.

**Reinforcement learning** Reinforcement learning maximizes task rewards by training agents to observe images and take actions, and can be applied to a variety of computer vision tasks such as object detection, image segmentation, and behavior recognition. The key to this approach lies in designing appropriate state space, action space, and reward functions for effective learning strategies. Man et al. [183] introduces a deep Q network (DQN) driven approach combined with a deformable U-Net architecture to address challenges in pancreas segmentation in medical image analysis, achieving accurate segmentation by interacting with contextual information and capturing geometry-aware features.

**Instance Segmentation** Dogan et al. [184] combined semantic segmentation and instance segmentation and proposed a two-phase approach. The first stage is Pancreas Localization, detecting the rough pancreas position on 2D CT slices by adopting the Mask R-CNN model. The second phase, Pancreas Segmentation, used the 3D U-Net model to refine the candidate pancreas region on 2D sub-CT slices.

**Figure 11:**
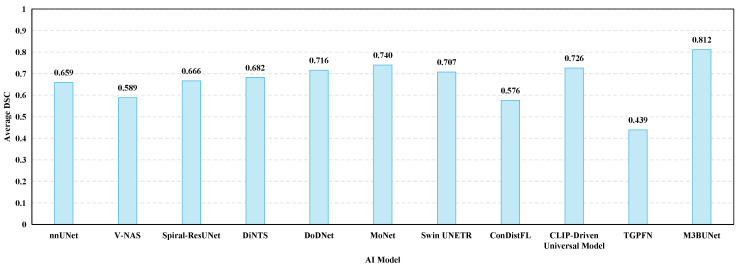
Summary of AI models’ segmentation performance for pancreas and PCs on MSD.

Figure 11 shows the average DSC of the pancreas and PCs for AI models in the MSD dataset from 2018 to 2024. AI models are sorted chronologically from left to right. While the overall performances improve over time, they still lag behind other organs.

**Figure 12:**
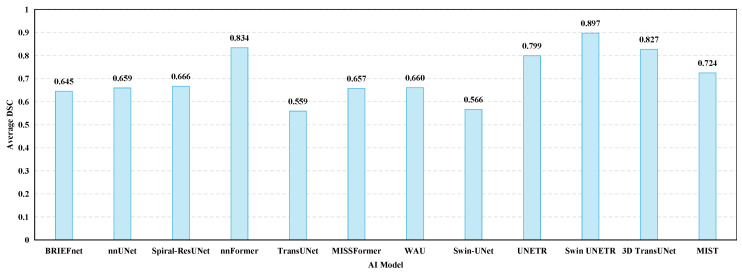
Summary of AI models’ segmentation performance for pancreas on BTCV.

Figure 12 shows the DSC of the pancreas for the AI models on the BTCV dataset from 2017 to 2024. AI models are sorted in chronological order from left to right. 3D segmentation models showed superior results.

Table 5 shows the comparison of AI models in CT pancreatic images for the segmentation task.

### 6.4. Object Detection

There are also several object detection works in CT images. Zhang et al. [185] proposed a pancreatic tumor detection framework that incorporated augmented feature pyramid networks, self-adaptive feature fusion, and a dependencies computation module. The framework also leveraged contextual information at multiple scales to improve detection accuracy. Baumgartner et al. [186] proposed nnDetection, a self-configuring method based on Retina U-Net [187] that could be deployed on arbitrary medical detection tasks. Juneja et al. [188] introduced a region-based CNN (RCNN)-crop method inspired by the region proposal network (RPN) and feature pyramid network (FPN). This approach extracts a cropped patch of the pancreatic region of interest (ROI) from CT images to promote accurate detection of PC. Dinesh et al. [189] proposed a novel YOLO model-based CNN (YCNN) for predicting PC in medical images. Their model utilized the YOLO architecture and CNNs to achieve efficient and accurate detection of pancreatic tumors.

Table 6 shows the comparison of AI models in CT pancreatic images for the object detection task.

### 6.5. Prognosis Prediction

Yao et al. [160] developed a 3D contrast-enhanced convolutional long short-term memory network (CE-ConvLSTM) that leverages tumor-vascular relationships for predicting the OS of PDAC patients. Zhang et al. [190] introduced a risk score-based feature fusion technique that integrated radiomics and transfer-learning features to improve the OS prediction performance for PDAC patients. Lee et al. [191] utilized ensemble learning to combine clinical data-based machine learning models (RF, GB, LR, NN, and SVM) and CT data-based deep learning models (3D ResNet-18 [192], R(2 + 1)D-18 [192], 3D ResNeXt-50 [193], and 3D DenseNet-121 [193]), leveraging preoperative data to predict postoperative survival. Chen et al. [194] developed a dual-transformation-guided contrastive learning scheme that effectively addressed data limitations and achieved excellent performance in predicting lymph node metastasis in PC.

Table 7 shows the comparison of AI models in CT pancreatic images for the prognosis prediction task.

### 6.6. Other Tasks

**Image reconstruction/denosing/super-resolution** Lyu et al. [196] reviewed 47 patients with pathologically confirmed PC who underwent baseline multiphasic CE-CT scans and used deep learning method for reconstruction, which enhances spatial resolution and reduces noise texture, improving accuracy in predicting PC resectability and reducing interreader variability while optimizing the tradeoff between spatial resolution and image noise in thin-slice CT images. Noda et al. [197] reconstructed pancreatic low-dose CT using deep learning image reconstruction and compared them with those of images reconstructed using hybrid iterative reconstruction. Chi et al. [198] proposed a Low Dose CT image super-resolution network that addresses spatial resolution loss and artifacts. It featured a dual-guidance feature distillation backbone containing a dual-guidance fusion module (DGFM) and a sampling attention block (SAB) and introduced the denoising head before and after the super-resolution head in each path to suppress residual artifacts. Takai et al. [199] found that deep learning based reconstruction substantially decreased background noise and enhanced both signal-to-noise ratio and contrast-to-noise ratio in pancreatic protocol CT scans at 80 kVp. Additionally, the highest quality and visibility of PDAC were achieved with the high-strength level of the deep learning reconstruction method. Shi et al. [200] proposed SR4ZCT, a self-supervised method that uses off-axis training to handle various combinations of resolution and overlap, explicitly modeling the relationship between resolutions and voxel spacings to accurately simulate training images matching the original through-plane images.

**Image generation** Liu et al. [201] trained a self-attention cycleGAN based on cone-beam CT (CBCT) acquired prior to the first fraction of treatment from thirty patients previously treated with pancreas SBRT to generate synthetic CTs. CT-based contours and treatment plans were then compared between first-fraction CBCTs and synthetic CTs. Similarly, Dai et al. [202] used cycleGAN to generate synthetic CT images from given CBCT images then trained the mask-scoring regional CNN (MS R-CNN) on generated images for segmentation. Shi et al. [203] introduced 3DGAUnet, utilizing GANs to produce realistic 3D CT images of PDAC. Its integration of a 3D U-Net architecture enhances the learning of shape and texture, improving efficiency and accuracy by preserving contextual information between slices, validated across diverse datasets, offering a promising solution to address data scarcity. Hooshangnejad et al. [204] developed a generation model named deepPERFECT that can capture minor differences and generate deformation vector fields to transform diagnostic CT into preliminary planning CT of PC, avoiding harm to patients because of separate image acquisition. Peng et al. [205] used TranscycleGAN to synthesize CECT from NECT and augment the amount of CT images. All real and synthesized CT images were used to train the modified 3D U-Net for the automatic delineation of gross tumor volume. Guan et al. [206] proposed a texture-constrained multichannel progressive GAN (TMP-GAN), using joint training of multiple channels. An adversarial learning-based texture discrimination loss is used to further improve the fidelity of the synthesized images and a progressive generation mechanism to improve the accuracy of the image synthesizer. Experiments of generating pancreatic tumor CT images were conducted.

## 7. Magnetic Resonance Imaging (MRI)

### 7.1. Introduction to MRI

Magnetic resonance imaging (MRI) is a noninvasive medical imaging technique that uses nuclear magnetic resonance (NMR) to create detailed, high-contrast, three-dimensional images of the body for diagnostic purposes [207,208,209]. Unlike X-rays or CT scans, MRI uses nonionizing radiation. It produces excellent contrast images of both soft and hard tissues, by utilizing static and slowly varying magnetic fields and electromagnetic energy in the high to very high-frequency bands [207]. Compared to typical CT scans, MRI provides superior contrast images. MRI scans are generally more time-consuming, taking 20 to 90 min depending on the body part being imaged, but they are painless and do not cause tissue damage [210].

However, MRI is not suitable for patients with certain metallic implants due to its reliance on magnetic fields and electromagnetic energy [207]. It is also important to note that MRI tends to be relatively more expensive. Nevertheless, despite these limitations, MRI remains an important tool in clinical diagnosis, providing crucial anatomical and pathological information to assist physicians in making accurate diagnostic and therapeutic decisions. As technology continues to advance, MRI may further improve its imaging speed and expand its range of applications, offering patients more accurate and convenient diagnostic services.

### 7.2. Classification

**Feature extraction + machine learning** Cui et al. [211] applied LASSO regression to classify low and high-grade branching type IPMNs (BD-IPMNs). They determined ROIs with radiologists and extracted features including histograms, texture parameters, RLM (run length matrix) GLCM and form factor parameters using MITK software (Medical Imaging Interaction Tookit 3.1.0.A, GE Healthcare). A linear combination of selected features with weights was used for grade prediction.

**End-to-end deep learning** Chen et al. [212] introduced PCN-Net for distinguishing between MCNs and SCNs in T2 and T1 weighted MRIs. The backbone of this framework utilized a pretrained InceptionV3 [213]. The fusion of the two modalities was achieved through a fusion algorithm, followed by a voting algorithm to obtain the results. In another study, Chen et al. [214] proposed a weighted loss function and applied it to various CNNs. It is proved that this weighted loss function could improve the accuracy of most CNNs and reduce the false negatives.

**Deep features + machine learning** Corral et al. [215] employed a pretrained (fast) CNN-F [216] to extract features from MRI images, resulting in formed vectors. These vectors were subsequently transformed using canonical correlation analysis (CCA) and fed into an SVM classifier. The SVM effectively classified the images into three distinct types: healthy pancreas, low-grade IPMN, and high-grade IPMN with PDAC.

**Unsupervised learning** Semi-supervised, weakly supervised, and unsupervised methods in machine learning and deep learning provide cost-efficient and scalable solutions by leveraging partially labeled or entirely unlabeled data. Hussein et al. [217] tried both supervised and unsupervised learning methods. 3D CNN with multitask learning was used as a supervised method. For unsupervised learning, they employed a proportion-SVM to classify IPMNs and normal pancreas. They initially cluster appearance features from images to estimate labels, then compute label proportions for each cluster, and finally use these initial assignments and proportions to learn tumor categorization.

### 7.3. Segmentation

**FCN/UNet-based methods for 2D segmentation** Asaturyan et al. [218] used a Hausdorff-Sine loss function to address vague organ boundaries in high class-imbalanced data, optimizing boundary delineation using the modified Hausdorff metric and a sinusoidal component in medical segmentation. Chen et al. [152] proposed the Spiral-ResUNet, which incorporated a spiral transformation to enhance segmentation performance. This UNet-based framework leveraged the residual block of ResNet-34 in the encoder module, enabling effective feature extraction.

**FCN/UNet-based methods for 3D segmentation** Liang et al. [219] involved registering MRIs, pre-processing, patch extraction, classification with a square-window-based CNN architecture, and post-processing to obtain a binary map representing tumor probability distribution, from from original T1-weighted DCE MRI. Li et al. [220] introduced a registration-free multimodal and multiscale adversarial segmentation network (MMSA-Net). This innovative network eliminated the need for registration between different modalities and scales by employing a shared encoder and two separate decoders. Mazor et al. [221] proposed an MC3DU-Net, which utilized TSE MRI scan for pancreas ROI segmentation, transferring it to MRCP scan for cyst detection and segmentation within the ROI, employing 3D U-Nets trained with Hard Negative Patch Mining to address class imbalance and reduce false positives.

**Graph-based methods for medical image segmentation** Cai et al. [222] conducted pancreatic detection and boundary segmentation using two CNN models: for tissue localization to differentiate pancreas and nonpancreas tissue based on spatial intensity context, and for boundary determination to delineate the semantic boundaries of the pancreas. The results from both networks are fused to initialize a conditional random field (CRF) framework, yielding the final segmentation output. Li et al. [223] proposed an end-to-end unsupervised domain-adaptive (UDA) segmentation method. This approach took advantage of GCN and a meta-learning strategy to address the challenges of adapting to target domains without labeled data.

### 7.4. Object Detection

Chen et al. [212] developed a three-stage modified Faster-RCNN approach. Firstly, they employed a pretrained VGG16 [224] to extract features from the input. These features were then used to identify the ROI. Subsequently, a Z-Continuity Filter (ZCF) was applied to filter the ROIs and improve the accuracy of the detection process.

### 7.5. Prognosis Prediction

Han et al. [225] applied logistic regression analysis and Cox proportional hazards regression to figure out the risk factors related to recurrence and disease-free survival (DFS) among pNET patients who had previously undergone surgery. They considered various MRI features such as size, location, margin, etc. The analysis revealed that certain MRI features, including portal phase iso-to hypoenhancement, dilatation of the common bile duct or main pancreatic duct, arterial invasion, and larger size, had a significant impact on poor DFS. In another study, Xu et al. [226] extracted MRI features by data-characterization algorithms in patients with PDAC. Then, the LASSO algorithm was utilized to calculate risk scores based on MRI features. Then, Cox proportional hazards regression was performed to create a radiomics-based nomogram to predict survival in patients with PDAC that combined radiomics data, clinical data and TNM information [227].

### 7.6. Other Tasks

**Image reconstruction/super-resolution** Chaika et al. [228] used deep learning-based super-resolution gradient echo imaging to enhance MRI image quality and reduce acquisition time for pancreatic imaging, minimizing artifacts and easily integrating into post-processing workflows without protocol modifications.

Table 8 shows the comparison of AI models in MRI pancreatic images for the classification, segmentation, object detection, and prognosis prediction task.

## 8. Endoscopic Ultrasonography (EUS)

### 8.1. Introduction to EUS

Endoscopic Ultrasonography (EUS) is a medical procedure that combines endoscopy and ultrasound technology to provide high-resolution imaging and detailed tissue characterization of the gastrointestinal tract and adjacent organs. It allows for the visualization of the digestive system’s walls and nearby structures like the liver, gallbladder, and pancreas. EUS has the ability to perform fine-needle aspiration (FNA), enabling tissue samples to be collected for analysis. This minimally invasive and well-tolerated procedure is especially effective in staging malignancies and evaluating pancreatic and biliary disorders, making it an invaluable tool for diagnosing and managing various gastrointestinal conditions.

EUS has demonstrated its superiority in detecting masses compared to CT scans. Studies have shown that EUS exhibits higher sensitivity in mass detection [229]. This improved sensitivity can be attributed to the close-range imaging capability of EUS, allowing for detailed examination and precise localization of abnormalities. Unlike conventional transcutaneous ultrasound examinations, EUS is not limited by pulmonary or bowel gas interference, ensuring accurate visualization and assessment of the pancreas in real-time. It provides high-resolution ultrasound images, enabling clinicians to identify and evaluate pancreatic lesions with exceptional clarity [230].

### 8.2. Classification

**Feature extraction + machine learning** Ruano et al. [231] focused on identifying interest points and calculating intensity gradients, resulting in 64 features from EUS images, which were used to create a frame feature vector for analysis and classification. To distinguish between PC and non-PC cases, the authors applied SVM and AdaBoost algorithms. Notably, their results outperformed deep learning methods in noisy experiments.

**End-to-end deep learning** Kuwahara et al. [232] employed ResNet-50 to predict the malignant probability of IPMN, the precursor of PDAC. The accuracy of this approach was higher than the human diagnosis. Zhang et al. [233] proposed a system called BP MASTER, which utilized ResNet in EUS videos to classify pancreas stations. Udriștoiu et al. [234] combined CNN and long short-term memory (LSTM) to classify PDAC, PNET, and chronic pseudotumoral pancreatitis (CPP) in EUS images. Nguon et al. [235] used ResNet-50 in EUS images for MCN and SCN classfication. Bonmati et al. [236] developed a CNN composed of two branches for voice data and image data, respectively, used to predict image labels from the spoken names of anatomical landmarks. Vilas et al. [237] applied the Xception model with pretrained weights to classify Mucinous and Non-Mucinous pancreatic cystic lesions. Jaramillo et al. [238] used GoogleNet, ResNet-18, and ResNet-50 to distinguish PC and non-PC classes. Ren et al. [239] used ResNet-50 with a feature fusion layer to combine with clinical features to classify three types of solid pancreatic tumors in EUS images: PDAC, pNET, and SPN. Kuwahara et al. [240] applied EfficientNetV2-L [241] to categorize various types of pancreatic tumors, including PDAC, pNET, SPN, PASC, ACC, metastatic pancreatic tumor, neuroendocrine carcinoma, chronic pancreatitis, and autoimmune pancreatitis. Fleurentin et al. [242] used different CNNs and ViT models to classify pancreatic anatomical landmarks and explored the effect of LSTM modules to utilize temporal information. Li et al. [77] introduced a Dual Self-supervised Multi-Operator Transformation Network (DSMT-Net), for multisource EUS diagnosis, which standardized region of interest extraction and employed a transformer-based dual self-supervised network for pretraining representation models using unlabeled EUS images.

Table 9 shows the comparison of AI models in EUS pancreatic images for the classification task.

### 8.3. Segmentation

**FCN/UNet-based methods for 2D segmentation** Zhang et al. proposed a system named BP MASTER (pancreaticobiliary master) [233] that employed a UNet++ to segment pancreatic boundaries and achieved comparable results to experts. Iwasa et al. [243] utilized UNet on contrast-enhanced EUS video images to investigate the influential factors in segmentation. They found that unclear tumor boundary (TB) negatively impacted the concordance rate, while respiratory movement (RM) had no significant effect. Oh et al. [244] employed the Attention U-Net model for automatic pancreatic cyst lesion segmentation and compared results with the Basic U-Net, Residual U-Net, and U-Net++ models. Seo et al. [245] developed DAF-Net (neural network model with deep attention features), which exhibited high accuracy and aided in effective surgical therapy for PC. Ren et al. [239] introduced an Attention UNet with a feature fusion layer for segmenting solid pancreatic tumors, assisting doctors in judging tumor scope and boundaries. Tang et al. [246] designed CH-EUS MASTER based on UNet++ with ResNet-50 as the backbone, a real-time capture and segmentation model for solid pancreatic masses using CH-EUS. The system offered equivalent tumor segmentation capabilities to trainer guidance. Studies showed that segmentation on EUS mainly relies on FCN architectures like UNet and exploration models with attention mechanisms. There have not been many attempts to use new techniques that can be further researched, like Transformer-based and other state-of-the-art methods.

Table 10 shows the comparison of AI models in EUS pancreatic images for the segmentation task.

### 8.4. Object Detection

There have been some works of object detection in EUS images or videos. Meyer et al. [247] introduced a real-time framework named the SELSA-TROIA model. It incorporated the sequence level semantics aggregation (SELSA) [248] and the temporal ROI align (TROIA) operator [249]. The SELSA considered the sequence information and aggregated features while the TROIA extracted temporal information. This framework simplified the procedure by identifying anatomical landmarks and addressing the time-consuming nature of mastering EUS. Tian et al. [250] applied YOLOv5m to EUS images and results showed promising real-time outcomes in detecting PC and reducing misdiagnosis. Jaramillo et al. [251] proposed a method to approximate the location of tumoral masses in conventional B-mode Echoendoscopy frames combining a dedicated classifier and an object detection YOLO architecture.

Table 11 shows the comparison of AI models in EUS pancreatic images for the object detection task.

### 8.5. Other Tasks

**Image Generation** Grimwood et al. [252] trained a Cycle-Consistent Adversarial Network with unpaired EUS images and CT slices extracted in a manner such that they mimic plausible EUS views, to generate EUS images from the pancreas, aorta, and liver, which can be used as a data augmentation strategy when EUS data is scarce.

## 9. Positron Emission Tomography (PET)

### 9.1. Introduction to PET

Positron Emission Tomography (PET) is an advanced nuclear imaging technique that utilizes radionuclides. PET provides information on the functioning of biological processes using radiolabeled tracers and quantitative mapping [253]. Fluorine-18 (F-18), Carbon-11 (C-11), Nitrogen-13 (N-13), and Oxygen-15 (O-15) are key positron-emitting radioisotopes employed in PET [254]. These isotopes enable the visualization and analysis of metabolic processes and find wide applications in diagnosing and treating various malignancies. PET has an advantage over conventional imaging techniques like CT and MRI since it can detect abnormal metabolic activity even without visible structural abnormalities in organs. This makes PET a powerful tool for early detection and monitoring of cancers. It is also valuable for post-treatment evaluation in cancer patients undergoing chemotherapy or tumor resection surgeries, assisting in assessing treatment response and the possibility of recurrence [255].

However, a challenge of PET is to precisely locate functional abnormalities within anatomical structures. This limitation has been addressed by integrating PET with high-resolution anatomic imaging modalities to form new images, such as PET-CT and PET-MRI. By merging the functional information from PET with detailed anatomical images, clinicians can accurately correlate metabolic activity with specific anatomical locations. This integration has significantly improved the diagnostic accuracy and clinical usefulness of PET in oncology [255,256,257]. The introduction of PET-CT in the early 2000s marked a significant milestone, enabling comprehensive and multimodal imaging that has greatly influenced the growth of oncology practices [256].

### 9.2. Classification

**Feature extraction + machine learning** Li et al. [258] presented the hybrid feedback-SVM-RF (HFB-SVM-RF) model, which incorporated 5 different kernels (Linear, MLP, Quadratic, Polynomial, and RBF) and 3 hyperplane separation methods (QP, SMO, LS) to construct a classifier. The features used in the model were extracted through dual threshold principal component analysis (DT-PCA), which combined principal features and nonprincipal features. Zhang et al. [259] extracted 251 expert-designed features from 2D and 3D PET/CT images of 111 patients and used RF, Adaboost, SVM with the Gaussian radial basis function kernel function (RBF SVM), and SVM with the linear kernel function (Linear SVM) to differentiate AIP from PDAC. Xing et al. [260] employed the XGBoost algorithm to analyze ^18^F-FDG PET-CT images for preoperative classification of PDAC into grade 1 and grade 2/3. Initially, the physicians manually segmented the ROIs. Pyradiomics [261] was used to extract radiomics features from the original images and the ROIs. Following this, the XGBoost model was built using the selected features to classify PDAC into grade 1 and grade 2/3.

**Feature extraction + deep learning** Zhang et al. [262] utilized a UNet encoder to extract image features and an RF algorithm to select important clinical features. Subsequently, they proposed a Trusted Multi-view Classification (TMC) algorithm to classify images as either low-grade or high-grade. Specifically, the term “low-grade” encompassed highly, moderately-highly, and moderately differentiated pathologies, whereas the “high-grade” category included undifferentiated, lowly, and moderate-lowly differentiated pathologies. Although clinical features were processed by RF, the image features were all processed in deep nets, and we regard the segmentation stage as a feature extraction process, thus we still categorized them in “feature extraction + deep learning”.

**Deep learning + machine learning** Wei et al. [263] combined deep features and radiomics features from PET and CT, which were fed into the RAD_model (the fully connected layers), the DL_model (the VGG11 network) and the MF_model (the fully connected layers), to classify PDAC and AIP.

### 9.3. Segmentation

**Superpixel extraction** Li et al. [258] developed a method called simple linear iterative clustering (SLIC) with the gray interval mapping (GIM) technique to convert CT scans into pseudo-color images. They then employed a combination of phase and frequency spectrum analysis to detect hypermetabolism areas in PET images.

**FCN/UNet-based methods for 2D segmentation** Zhang et al. [262] improved the UNet model by incorporating guidance from organ location and applying post-processing techniques such as erosion, expansion, and threshold segmentation (OLP).

**FCN/UNet-based methods for 3D segmentation** Suganuma et al. [264] used DenseUNet for multiple organs including pancreas segmentation combining information from PET and CT images. Wang et al. [265] introduced the multimodal fusion and calibration networks (MFCNet) for segmenting three-dimensional PET-CT images. Their framework included a multimodal fusion down-sampling block (MFDB) with a residual structure that fused features from various modal images. Additionally, they employed a multimodal mutual calibration block (MMCB) based on the inception structure, which combined decoding features and pathological features. Shao et al. [266] combined a cross multimodal fusion (CMF) module with a cross-attention mechanism to fuse complementary multimodal features, while a mutual information minimization (MIM) module mitigates redundant high-level modal information and computes the latent loss of PET and CT, enabling effective feature extraction and segmentation of regions of interest from PET/CT images using a semi-supervised framework.

### 9.4. Object Detection

Wang et al. [267] proposed the Multi-scale adaptive attention feature fusion (MAFF) network for tumor detection in PC using PET-CT imaging, which combined PET and CT strengths to improve accuracy. The network used a feature pyramid module for multiscale feature extraction, an attention module for feature screening, and an adaptive attention feature fusion network for selecting semantic information.

### 9.5. Prognosis Prediction

Park et al. [268] used a semi-automatic gradient-based method to determine volumes of interest (VOI). They applied LASSO regression to extract clinical and radiomic features from these VOIs. Finally, a 100-layer NN was employed to predict the progression of the disease within two years for patients with pNET.

Table 12 shows the comparison of AI models in PET pancreatic images for the classification, segmentation, and object detection task.

## 10. Pathological Images

### 10.1. Introduction to Pathological Images

Pathological images (or histopathological images) serve as visual representations of tissue samples observed through a microscope, playing a pivotal role in medical diagnosis, research, and treatment planning. Pathologists rely on these images to detect anomalies, characterize diseases, and provide guidance to clinicians.

The advent of computer-assisted diagnosis (CAD) in the 1990s revolutionized medical imaging and diagnostic radiology, concurrently reducing the workload of pathologists [269]. Digital pathology enables the digitalization and analysis of these images, leading to improved diagnostic accuracy [270]. At the core of digital pathology lies whole slide imaging (WSI), a technology that converts camera-captured static images into a digital format. WSI entails the scanning of slides via a scanner, followed by the analysis of resulting digital files using specialized software. In research, pathological images are invaluable for studying disease mechanisms and developing targeted therapies [271]. By integrating with other clinical data, they enable a comprehensive understanding of diseases and support personalized medicine. Ultimately, these images are indispensable in medical practice and contribute to advancing patient care.

Rapid on-site evaluation (ROSE) is a diagnostic technique that uses fine-needle aspiration (FNA). ROSE is of critical importance in the extraction of samples from deeply seated organs by nonsurgical means. Furthermore, it maintains an intrinsic connection with pathology by providing images and facilitating the evaluation of masses [272,273].

### 10.2. Classification

**Feature extraction + deep learning** Saillard et al. [274] proposed a deep learning-based approach named PACpAInt that accurately identifies tumor cell types and molecular phenotypes from routine histological slides, enabling comprehensive analysis of intratumor heterogeneity on a large scale and providing independent prognostic value.

**End-to-end deep learning** Chang et al. [275] proposed a deep learning based nucleus classification (DeepNC) method using CNN to classify cancerous and normal cells at a single-cell level. Le et al. [276] presented the Noisy Label Classification (NLC) method, also known as the NLC model, utilizing patches from WSIs to classify regions as cancerous or noncancerous. Sehmi et al. [277] used 14 different CNN models with pretrained models on ImageNet for PC grading in pathological images. Ono et al. [278] utilized CNN to extract features from ROSE (Rapid On-site Evaluation) images and proposed Information-Maximizing Self-Augmented Training (IMSAT) based on these features, resulting in highly accurate cluster analysis. The clustering results revealed distinct differences in features and cell density among different categories. Zhang et al. [279] introduced the Shuffle Instances-based Vision Transformer (SI-ViT) model, which effectively reduced perturbations in ROSE images, leading to significant improvements in performance. Ghoshal et al. [280] presented a Bayesian CNN for automated PC grading from MGG and H&E stained images to estimate the uncertainty in model prediction. They analyzed the relationship between the accuracy and uncertainty, and leveraged uncertainty in classification error and reject tradeoff. Kou et al. [281] proposed a hybrid CNN-Transformer model incorporating deformable atrous spatial pyramids (DACTransNet), performing automated and accurate classification of histopathological images of PC.

Table 13 shows the comparison of AI models in pathological pancreatic images for the classification task.

### 10.3. Segmentation

**FCN/UNet-based methods for 2D segmentation** In the study of Janssen et al. [282], a single H&E-stained slide of resected PC post-NAT from 64 patients was digitized, manually segmented into the tumor, normal ducts, and remaining epithelium classes, with resulting masks and patches distributed across training, validation, and test sets. Modified U-Nets employing different encoders were trained, achieving the highest mean segmentation accuracy with a DenseNet161 encoder. Yang et al. [283] proposed a selective multiscale attention (SMA) block for gland segmentation in the pancreas, featuring a selection unit between the encoder and decoder to amplify effective information and suppress redundant information based on a training-derived factor. Fu et al. [284] applied UNet for PDAC segmentation in WSIs. Gao et al. [285] put forward a selected multiscale attention network (SMANet) to accomplish tumor cell segmentation, incorporating the selection unit (SU) module and the multiscale attention (MA) module, effectively enhancing feature filtration and information supplementation. Zhang et al. [286] developed a DCNN system based on UNet for rapid on-site cytopathology evaluation (ROSE) to improve the diagnosis efficiency. This system demonstrated exceptional robustness and generalization ability. Liu et al. [287] introduced the multilevel aggregation and global guidance network (MLAGG-Net). Gao et al. [288] devised a multitask learning framework that adopted the EfficientNet-b0 encoding structure, featuring mobile inverted bottleneck convolution (MBConv) with squeeze-and-excitation (SE) modules to extract image features efficiently. Output utilizes a hierarchical sharing design, with three pathways designed for the main task and two auxiliary tasks, sharing more parameters as task correlation increases. Chen et al. [289] introduced a channel-spatial self-attention module, adaptable for mainstream segmentation networks, enhancing long-range dependency in feature maps and improving segmentation performance in PC pathology image segmentation.

Table 14 shows the comparison of AI models in pathological pancreatic images for the segmentation task.

### 10.4. Other Tasks

**Image Super-resolution** Li et al. [290] reconstructed high-resolution histology images from low-resolution inputs, employing multiscale FCN to capture hierarchical features and integrate conditional generative adversarial loss to mitigate blurriness in output images. Tissue microarray (TMA) dataset used in experiments was previously used in published PC studies.

**Image Reconstruction** Kugler et al. [291] proposed a fully nonrigid image registration method for 3D reconstruction of a whole PC Tumor from Pathology Images with different stains, considering the spatial continuity and smoothness of each constituent part of the microstructures in the tissue. They further proposed a nonrigid 3D reconstruction method based on smooth and continuous internal tissue assumptions. Landmarks detected via template matching with NCC form trajectories across slices, smoothed during registration, while NCC confidence handles artifacts by rejecting unreliable landmarks [292]. Although these two works were all done on the pancreas of KPC mice, they can also be referred to in research on human beings.

## 11. Multiple Modalities Analysis

Several studies have employed multiple types of medical images in their AI models. Combining various modalities effectively augments the dataset. This process enables AI models to gain visual information on PC tissues from different modalities, consequently improving accuracy by compensating for individual modality limitations, and creating more robust and discriminative feature representations, just as doctors sometimes need to perform multiple imaging examinations to make a diagnosis. Especially, integrating imaging with pathological data provides a more comprehensive and in-depth understanding at both microscopic and macroscopic levels. Combining imaging with pathological data bridges the information gap between the two, facilitating more accurate and detailed diagnosis and treatment planning. Similarly, combining various MRI modalities enhances accuracy by providing complementary perspectives. Structural imaging offers anatomical details, diffusion-weighted imaging detects tissue changes, and functional MRI reveals brain activity. Analyzing these together improves diagnostic precision and physiological understanding.

### 11.1. Traditional Machine Learning

In traditional machine learning, using features from multiple modalities of images as input is a common practice, which enhances model performance by combining diverse information to capture richer patterns and relationships in the data, improving predictions or classifications. Panda et al. [293] leveraged PET-MRI and CT metrics to predict OS. Principal component analysis was used to extract CT textural features while intra-class correlation, and the Cohen kappa correlation coefficient were used to extract PET-MRI features, then a Cox proportional hazards regression to predict OS using these features. Koch et al. [294] employed CT and MRI images to classify malignant tissue and predict all-cause mortality. In their study, radiologists initially performed semi-automatic segmentation and feature extraction on CT images using the GrowCut algorithm. Following this, they used Cox proportional hazards regression to predict the survival time of patients from the time of imaging until death from any cause.

### 11.2. Muti-Modal Fusion

Methods of multimodal fusion include feature-level fusion and decision-level fusion. Feature-level fusion combines feature vectors from different modalities into a larger feature vector, typically achieved by concatenation or concatenation. Decision-level fusion combines independent decisions or predictions from different modalities, such as through voting or weighted averaging.

Feature-level fusion involves extracting features from each modality, normalizing and aligning them if necessary, and then combining them into a single, fused representation. This fused representation captures information from multiple modalities and can be used as input for subsequent machine learning tasks. Feature-level fusion often relies on specially designed feature fusion modules. Attention-based fusion methods are typical feature-level fusion techniques, allowing models to dynamically weight the contribution of different modalities or regions within modalities based on their relevance to the task at hand. This selective weighting helps in enhancing the discriminative power of the fused representation while suppressing noise or irrelevant information.

Hussein et al. [295] proposed a CNN-based CAD system for IPMN diagnosis and risk assessment using multimodal MRI, employing minimum and maximum intensity projections to mitigate annotation variations and a CNN to extract deep features from T1-weighted and T2-weighted MRI modalities. Finally, canonical correlation analysis (CCA) is utilized for feature-level fusion to derive discriminative canonical correlation features, which are then employed for classification. In the fusion part of Chen et al.’s works [214], they arranged all slices with an ROI into a volume (simply jump the blank slices when testing) with their Z-axis index, then resampled the modality with fewer slices to increase the number of slices. As a result, images of two modalities have the same number for every patient. Chen et al. [296] introduced a model-driven multimodal deep learning approach, using a spiral transformation algorithm to convert 3D data into 2D images, preserving spatial correlation and edge information. The prior knowledge for multimodal fusion was introduced, enhancing performance, particularly with small sample sizes. Zhang et al. [297] developed a multimodal fusion system Asymmetric Twinning Information Interaction Network (ATIIN) to predict the postoperative survival time of PDAC patients by utilizing both CT images and WSIs. In the ATIIN system, CT images and WSIs were processed by ResNet-101 and ResNet-50, respectively. Subsequently, feature and channel attention techniques were applied before combining the features to obtain the final results. Their study combined the advantages of radiomics and pathomics and improved the cost-benefit ratio of PCs.

### 11.3. Cross-Modality Transfer Learning

Cross-modal transfer learning is widely applied in medical imaging to enhance image recognition, segmentation, and feature extraction tasks by transferring knowledge from one modality to another, improving diagnostic accuracy and reducing the need for labeled data. It exploits correlations and shared information between different modalities, mitigating the challenges of data scarcity and enhancing model generalization and robustness against noise and artifacts in medical images. Yao et al. [298] introduced the Transferred DenseSE-Mask R-CNN (TDSMask R-CNN) Network to segment pancreatic tumors, incorporating Dense and Squeeze-and-Excitation (SE) blocks to learn complementary features from both PET and MRI images. To overcome the challenge of limited labeled data in PC segmentation, they pretrained the Dense-SENet on PET images and then transferred its weights for MRI images.

### 11.4. Deep Learning-Based Image Modality Conversion

Deep learning-based image modality conversion offers the advantage of maximizing data utilization in medical imaging and reducing annotation burdens, especially in scenarios with limited data availability. Training on one modality and converting other modalities to the trained format for inference or converting different modalities into a unified format, streamlines processing workflows can improve model performance by adapting to more suitable modalities. Li et al. [299] generated random intermediate modalities between MRIs and CT to form a larger dataset. Then, they improved Res-UNet with meta-learning strategies. This framework could be easily integrated into other segmentation networks and alleviate data scarcity. Cai et al. [300] proposed a generic cross-modality synthesis approach using an end-to-end 2D/3D CNN, where mutually beneficial generators and segmentors collaborate for image synthesis and segmentation tasks. This method synthesizes realistic images without paired training data, maintains consistent anatomical structures, and improves segmentation performance by using synthetic data.

### 11.5. Multi Modality-Tasks Models

Some models are not only designed for a single modality but also aim to achieve good performance on multiple modal tasks. Cai et al. [301] propose a CNN-RNN model for pancreas segmentation in radiology images, aiming to improve segmentation accuracy by integrating adjacent slice information. The model combines a 2D CNN for initial segmentation with an RNN using CLSTM units for refining segmentation consistency across slices, achieving better performance on both CT and MRI images. Asaturyan et al. [302] introduced a 2D/3D method for pancreatic segmentation on multimodal radiological scans, which incorporates a novel post-processing stage to improve tissue classification through progressive contour analysis. The approach ensures detailed boundary preservation, spatial smoothness, and consistent tissue classification across slices, with potential applicability to other abdominal MRI and CT sequences and broader segmentation tasks.

Table 15 shows the comparison of AI models in multiple modalities analysis.

## 12. Tools, Frameworks, and Software

For intelligent analysis of PC medical imaging, not only methods or algorithms are important, but also the support of tools, frameworks, and software, which will provide great convenience for data annotation, algorithm development, clinical usage, and the integration of medical and engineering research.

### 12.1. Visulization and Annotation Tools

Medical image annotation is of paramount importance for training deep learning models, particularly due to the specialized expertise required for accurate labeling. Medical images often encompass complex structures and pathologies, necessitating precise annotations crucial for training deep learning models. In addition, annotations for medical images require high levels of accuracy to prevent misdiagnosis or erroneous treatment plans. Moreover, the 3D image annotation is even more important. Unlike 2D images, 3D images involve additional spatial dimensions, requiring more comprehensive annotation information.

Therefore, the significance of annotation and visualization platforms cannot be overstated. DicomWorks [303], free software for reading and working on medical images in DICOM format, offers several tools for analysis and annotation. The three-dimensional (3D) slicer [304] is a free, open-source platform for visualization, processing, segmentation, registration, and analysis of medical, biomedical, and other 3D images and meshes, widely used by researchers, clinicians, and developers for its interactive tools and stable platform, remaining compatible with the latest hardware and software advancements. It witnessed continuous development based on a 3D slicer from the joint effort of the community. ITK-SNAP [72], a freely available, open-source software tool designed for segmenting structures within 3D and 4D biomedical images. This versatile application offers semi-automatic segmentation capabilities utilizing active contour methods, alongside manual delineation and intuitive image navigation features. RIL-contour [305] allows using fully automated deep learning methods, semi-automated methods, and purely manual methods with voxel and/or text annotations. It uses iterative deep learning to accelerate annotation. In order to perform efficient semi-auto annotation on 3D medical images, EISeg-Med3D [306,307], a 3D slicer extension, is designed to help users guide a deep learning model to perform segmentation by providing positive and negative points.

User-friendly, efficient, interactive, semi-automatic medical image annotation tools will contribute to high-quality medical image datasets, research in medical deep learning algorithms, and algorithms related to PC diagnosis.

### 12.2. Platform, Software, and Packages of Radiomics

The platform, software, and packages for radiomics are essential as they enable the extraction, quantification, and analysis of radiomic features from medical images. They provide the infrastructure, tools, and algorithms necessary for efficient data processing, facilitating research and clinical applications. PyRadiomics [261] is an open-source Python package for extracting radiomics features from medical images, aiming to establish a reference standard for radiomics analysis, providing a tested and maintained platform for reproducible feature extraction. With support for both 2D and 3D analysis, it enables calculations of single values per feature for ROI or generation of feature maps. The Quantitative Image Feature Engine (QIFE) [308] is an open-source, modular system for 3D radiomics feature computation. It integrates seamlessly into existing workflows, focusing on modularity, standards, and parallelism. It offers both MATLAB code and a Docker container for easy deployment, with benchmarking showing significant time savings with parallelization. Researchers can customize components and optimize computational efficiency based on dataset characteristics.

### 12.3. Framework of Deep Learning Designed for Medical Image Analysis

Due to issues such as data reading formats in medical imaging, convenience is not always guaranteed. Frameworks specifically tailored for deep medical imaging, encompassing data reading, preprocessing, commonly used algorithm models, etc., not only enhance convenience but also facilitate fair comparisons of state-of-the-art methods. DLTK [309] is a toolkit based on TensorFlow developed to enable fast prototyping with a low entry threshold and ensure reproducibility in medical image analysis, containing several popular architectures of networks. However, it has not been updated for a long time. TorchIO [310] is an open-source Python library for preprocessing, augmentation, and sampling of medical images for deep learning, which supports 2D, 3D, and 4D images such as X-ray, histopathology, CT, ultrasound and diffusion MRI. MONAI [311] extends PyTorch for medical data, offering specialized AI model architectures, transformations, and utilities to simplify medical AI model development and deployment, which also maintains the simplicity and compositional nature of PyTorch libraries it builds upon. MedicalSeg [306,312], an easy-to-use 3D medical image segmentation framework handling the whole segmentation process including data preprocessing, model training, and model deployment based on PaddlePaddle deep learning framework. It supports many cutting-edge models and corresponding high-precision pretraining models. Although these frameworks provide users with great convenience, the constant emergence of new methods in academia and industry poses challenges for the timely updating of these unified frameworks.

## 13. Special Topics and Future Directions

### 13.1. Efficient and Light Model Design

Given the constraints of devices’ performance in hospitals, the importance of designing medical imaging models that are efficient and lightweight cannot be overstated. These models are tailored to operate seamlessly within the limitations of hospital hardware, ensuring swift and accurate processing of medical images without taxing computational resources. By prioritizing efficiency and minimizing computational overhead, such designs empower healthcare professionals to swiftly analyze medical images, facilitating timely diagnoses and enhancing patient care. Models and backbones like MobileNet [313] and ShuffleNet [314], as well as real-time detection models like tiny versions in the YOLO series, and lightweight U-Net variants like UNext [315], MALUNet [316] and EGE-UNet [317] are specifically designed to address computational efficiency. However, in PC analysis, there is still relatively little focus on lightweight design and real-time performance.

### 13.2. Domain Generalization

Although deep learning models have achieved comparable results to radiologists on specific datasets, the imaging equipment and pancreas morphology vary greatly in the real world. To realize the wide application of deep learning models for clinical diagnosis and treatment, the domain shift problem needs to be addressed, namely the distributional gap between training and test data. Domain generalization aims to solve this problem by developing models with stable performance for unknown domains. Differences in data acquisition, high-dimensional data, data labeling, and model ethics are challenges in domain generalization for medical image analysis [318].

Data-level domain generalization includes data manipulation and data augmentation. Data manipulation transforms the existing data, while data augmentation creates new samples based on the existing ones. In addition, methods are used to process particular input image modalities, like cross-modal generative models [319,320,321] and stain normalization [322]. Feature-level domain generalization utilizes domain-invariant features to improve model performance, feature alignments, disentanglement methods, feature augmentation, and kernel-based learning are commonly used techniques. Model-level domain generalization focuses on the improvement of learning strategy and model framework. Meta-learning, self-supervised learning, and adversarial learning are effective learning strategies. Ensemble learning, model distillation, and distributed learning are typical improved models. Zhang et al. [323] introduced a deep stacked transformation approach for domain generalization. During network training for 3D segmentation tasks on MRI and EUS images, a series of stacked transformers were applied to each image. Research on domain generalization of pancreatic medical images is limited, and universal AI models that can be used for clinical diagnosis and treatment still need to be developed from the direction of data, feature, model, and analysis levels.

### 13.3. Multimodal Tasks

Medical Visual Question Answering (MedVQA) is an AI technology designed to answer questions related to medical images. This technology combines computer vision and NLP, enabling computers to understand medical images and respond to questions about them. PMC-VQA [324], PathVQA [325], and VQA-RAD [326] are all good works as public VQA datasets. Although some public VQA datasets include questions related to the pancreas and PC, they are insufficient to cover the full spectrum of cases. There has not been specific medical VQA research dedicated solely to the pancreas and PC. This is an area ripe for exploration and offers potential for utilizing multimodal large models, through which doctors and researchers can query computers about patient diagnoses, treatment plans, and more about the health of the pancreas, obtaining answers from medical images, helping improve the efficiency and accuracy of medical diagnoses.

The report generation task in clinical images is close to MedVQA, automatically generating textual descriptions or summaries based on the content of medical images, such as X-rays, MRI scans, CT scans, and histopathology slides, which can assist radiologists, pathologists, and other medical professionals in interpreting and documenting findings from medical images efficiently. Related works about the pancreas and PC in images of different modalities are also limited, which can be further explored.

Recently, large multimodal language models (LLM) have achieved notable success in general domains but face limitations in medical scenarios due to significant differences between medical images and text. Currently, visual-language and multimodal models tailored to specific organs or diseases are also being developed, such as OphGLM [327], a newly developed ophthalmic multimodal model, demonstrating the potential for revolutionizing clinical applications in ophthalmology. Drawing inspiration from the progress in ophthalmology, the development of a dedicated pancreatic multimodal language model (PMLM) could also be expected.

### 13.4. Large Model Empowered Solutions

Large models’ increased capacity allows them to capture and understand complex patterns and relationships within data more effectively. Moreover, large models tend to have better generalization capabilities, adapting well to new, unseen data and domains. They enable more sophisticated and nuanced representations of information, facilitating more accurate and insightful outputs.

Contrastive Language-Image Pretraining (CLIP) [180] stands as a simple yet potent pretraining paradigm. Thanks to its versatility and interpretability, it demonstrates promising results across a spectrum of tasks. It also has gained increasing attention and achieved wide application in the field of medical image analysis, serving as a pretraining paradigm for image-text alignment, or a component in different clinical tasks [328] including zero-shot classification [329], object detection [330], 2D image segmentation [331] and 3D image segmentation [178,179], as well as some cross-modality tasks [332]. As CLIP continues to evolve and adapt to the specific challenges posed by medical image analysis, its integration into clinical practice in PC diagnosis and treatment is expected to grow exponentially.

As we mentioned earlier, the segment anything model (SAM) [333] has been trained on millions of images and more than a billion masks, enabling it to produce effective segmentation masks for any input, archiving impressive zero-shot performance. And experiments show that it can also be a valuable tool in medical image segmentation if used correctly [176]. The continuous effort to make SAM adapt to medical images or to train a new zero-shot medical image segmentation has been witnessed. Zhang et al. [334] proposed SAMed, applying the low-rank-based (LoRA) fine-tuning strategy to the SAM image encoder and fine-tuning it together with the prompt encoder and mask decoder on labeled medical image segmentation datasets. Wu et al. [335] proposed the Medical SAM Adapter (Med-SA), incorporating domain-specific medical knowledge into the segmentation model, using Space-Depth Transpose (SD-Trans) to adapt 2D SAM to 3D medical images and Hyper-Prompting Adapter (HyP-Adpt) to achieve prompt-conditioned adaptation. Ye et al. [336] introduced SA-Med2D-20M, a large-scale segmentation dataset of 2D medical images built upon numerous public and private datasets, which consists of 4.6 million 2D medical images and 19.7 million corresponding masks, covering almost the whole body and showing significant diversity to incorporate medical knowledge into SAM. Although there have been some efforts in this regard, achieving zero-shot segmentation of the pancreas and PC remains challenging due to the limited availability of datasets containing diverse modalities of pancreatic and PC data. Larger and more diverse datasets of PCs are expected, which will facilitate the ability of large models to better address PC-related challenges.

### 13.5. Explainability

Deep learning models often appear as black boxes, and medical experts have expressed concern about such a nature [337]. Healthcare professionals and researchers need to comprehend the model’s decision-making process and outcomes to ensure the reliability of the diagnosis and treatment decisions. In explainability research, most current works utilize post hoc explanation methods instead of model-based explanations, providing explanations on trained neural networks rather than incorporating them during training, predominantly employing local explanations rather than global ones, particularly suited for deep learning in medical image analysis. In the future, the adoption of holistic approaches, the integration of biological explanations, and the exploration of the link between causality and Explainable AI will become increasingly important [338]. Therefore, exploring the explainability of deep learning models in the pancreas and PC research could be a promising direction for future investigation.

## 14. Conclusions

This study summarized applications of AI on five modalities and integrated modalities of medical images related to the pancreas and PC. AI models demonstrate reasonable results in segmentation, classification, object detection, prognosis prediction, and other tasks in the experiment stage and perform similarly to human experts in many studies, which highlights the potential of AI to help doctors and alleviate their workload in the diagnosis and treatment of PCs. However, the overall accuracy of the pancreatic analysis lags behind that of other organs, mainly due to the size and variable characteristics of the pancreas. In addition, AI studies using MRI, pathology images, and PET imaging for target detection and prognosis prediction are still limited. The lack of comprehensive medical image datasets and further modeling studies challenges the widespread use of AI techniques in clinical settings. Despite these challenges, lightweight model design, multimodal tasks, large model-empowered solutions, and explainability are future directions that will enhance the efficiency and reliability of AI-based analysis. In addition, AI scientists should work more closely with doctors while also continuing to improve human understanding and attention to PC. With the joint efforts of all sectors, the probability of early screening for PC will be increased, and the threat posed by this disease to human health will be overcome as much as possible. 

## Figures and Tables

**Table 1:** Comparison of this paper with existing reviews in AI-enabled pancreatic image analysis.

Reference	Year	Brief Summary	AI Models in Pancreatic Imaging Processing	Multiple AI Tasks and Evaluation Metrics	Different Pancreatic Imaging Modalities	Future Directions for AI in PC Research
[17]	2019	A review on deep learning in the differential diagnosis of PC and CP	M	L	N	L
[18]	2020	A review on early detection of PC	L	L	M	N
[19]	2021	A summative review on PDAC early detection	H	L	M	N
[20]	2021	A comprehensive review on PC screening and diagnosis strategies	L	L	H	N
[16]	2022	A review on application of AI in PC diagnosis	H	H	M	M
[21]	2022	A review on AI in PC diagnosis based on medical imaging and biomarkers	H	L	H	N
[22]	2022	A systematic review on AI and machine learning in pancreatic surgery	M	L	M	H
[23]	2022	A review on AI in PDAC diagnosis and prognosis from CT images	H	H	N	H
[24]	2023	A scoping review on PC diagnosis and prediction using AI	M	M	N	M
[25]	2023	A narrative review on AI in PC diagnosis, biomarkers detection, and prognosis	L	L	M	M
[26]	2024	A review on AI in various aspects of PC	H	M	M	H
[27]	2024	A review on AI in PC early diagnosis	H	M	M	N
This paper	-	A comprehensive review on AI in pancreatic images processing	H	H	H	H

Depth of discussion: H—high, M—moderate, L—low, N—not discussed.

**Table 2:** Search terms.

Search Term	Set of Keywords
Pancreatic	pancreatic cancer, pancreatic lesion, pancreatic cancer diagnosis, pancreatic cancer detection, pancreatic ductal adenocarcinoma, pancreatic neuroendocrine tumors
Cancer	cancer subtypes, precursor lesions, cancer diagnosis, cancer treatment
AI task	classification, segmentation, object detection, prognosis prediction, image registration, image generation, super-resolution, denoising, reconstruction, medical visual question answering, natural language processing
Image modality	CT, MRI, EUS, PET, pathological images, PET/CT, multimodal fusion, multiple modalities, cross-modality, modality conversion
Machine learning	Cox proportional hazards regression, Logistic regression, least absolute shrinkage and selection operator regression, decision tree, support vector machine, random forest, ensemble learning, k-nearest neighbors, k-means clustering
Deep learning	convolutional neural networks, fully convolutional neural networks, transformers, recurrent neural networks, long short-term memory, you only look once, graph neural networks, federated learning, reinforcement learning, neural architecture search
Large model	contrastive language-image pretraining, segment anything model

**Table 3:** Features extracted by six methods in [130].

Methods	Feature Name
Shape	height, width, perimeter, area, complexity, rectangularity, elongation, equivalent area radius
GLCM	mean and standard deviation of energy, entropy, moment of inertia, and correlation
GLRLM	short run emphasis, long run emphasis, gray-level nonuniformity, run percentage, run-length nonuniformity, low gray-level run emphasis, high-gray level run emphasis
GLGCM	small grads dominance, big grads dominance, gray asymmetry, grads asymmetry, energy, gray mean, grads mean, gray variance, grads variance, correlation, gray entropy, grads entropy, entropy inertia, differ moment
GLDS	mean, contrast, angular second moment, entropy
Wavelet transform	

**Table 4:** Summary of AI methods in CT images for classification task.

Year	Reference	Model	Dataset	Sample Size	Performance
2020	[130]	LASSO regression and EL-SVM learner	A private dataset	168	AUC = 0.7308 (normal–early stage), 0.6587 (normal–stage III), 0.7333 (normal–stage IV)
2021	[131]	XGBoost	A private dataset, MSD and NIH	27,235, 5715, and 7054	AUC = 0.97 (private test set), 0.83, and 0.89 (public test set)
2022	[133]	KNN, SVM, RF and XGBoost	A private dataset and NIH	596 and 82	AUC = 0.95, 0.98, 0.95, and 0.96
2020	[11]	VGG	A private dataset, MSD and NIH	14,780, 4849, and 1427	Accuracy = 0.986, 0.989 (private test set), and 0.832 (MSD and NIH test set)
2021	[134]	UNet with Anatomy-aware Hybrid Transformers	A private dataset	1627	Recall = 0.952, Specificity = 0.958
2023	[135]	PANDA	Five private dataset	3208, 786, 5337, 18,654, and 4815	Specificity = 0.999, Recall = 0.929, AUC = 0.986–0.996
2022	[136]	IDLDMS-PTC	A private dataset	500	Accuracy = 0.9935, Specificity = 0.9884, Recall = 0.9935, F1-score = 0.9948
2023	[137]	DenseNet	NIH and MSD	18,942 and 15,000	Accuracy = 0.974, Specificity = 0.966, Recall = 0.983
2022	[138]	DNN-MMRF-ResNet	A private dataset	110	Precision = 0.9387, Recall = 0.9136, Specificity = 0.9380, Accuracy = 0.9269
2023	[139]	Stacking ensemble	NIH	80	Accuracy = 0.988

**Table 5:** Summary of AI methods in CT images for segmentation task.

Year	Reference	Model	Dataset	Sample Size	Performance
2015	[140]	SLIC	NIH	82	DSC = 0.81
2015	[67]	Probabilistic bottom-up approach	NIH	82	DSC = 0.805
2017	[141]	BRIEFnet	BTCV	30	DSC = 0.645
2017	[142]	FCN-8s with DSN	A private dataset	131	DSC = 0.6344±0.2771
2019	[144]	Ringed Residual U-Net	NIH	82	DSC = 0.8832±0.0284
2020	[145]	iUNet	A combination of TCIA and BTCV, and a private dataset	90 and 1905	DSC = 0.87
2020	[146]	DLU-Net	MSD and a private dataset	281 and 126	DSC = 0.9117 and 0.9094, Accuracy = 0.9725 and 0.9743
2020	[147]	Custom segmentation network	NIH	82	DSC = 0.8757±0.0326
2022	[148]	WAU	BTCV	30	DSC = 0.6601
2023	[149]	LMNS-net	NIH	82	DSC = 0.8868, IoU = 0.9882, Precision = 0.6822, Recall = 0.9866
2024	[150]	M3BUNet	NIH and MSD	82 and 281	DSC = 0.8952 and 0.8860, IoU = 0.8116 and 0.7990
2023	[151]	DBFE-Net	Two private datasets	116 and 42	Precision = 0.6573 (PCs), 0.8907 (abnormal) and 0.9147 (normal)
2023	[152]	Spiral-ResUNet	MSD	281	DSC = 0.6662
2018	[154]	3D UNet	A private dataset	147	DSC = 0.897±0.038
2019	[155]	CNN with Bias-dice loss function	NIH	82	DSC = 0.8522
2019	[156]	3D UNet-based two-stage framework	NIH	82	DSC = 0.8599
2021	[157]	DoDNet	MSD	281	DSC = 0.7155, HD = 11.70
2021	[158]	CNNs with STFFM and PPM modules	NIH and MSD	82 and 281	DSC = 0.8490 and 0.8556
2018	[159]	nnUNet	MSD	281	DSC = 0.659
2020	[160]	nnUNet	A private dataset	61	DSC = 0.73
2021	[98]	Transformer-UNet	NIH	82	mIoU = 0.8301, DSC = 0.7966
2021	[161]	MISSFormer	BTCV	30	DSC = 0.6567
2021	[96]	TransUNet	BTCV	30	DSC = 0.5586
2022	[97]	Swin-UNet	BTCV	30	DSC = 0.5658
2023	[162]	TD-Net	NIH and MSD	82 and 281	DSC = 0.8989 and 0.9122
2024	[163]	MIST	BTCV	30	DSC = 0.7243
2021	[164]	nnFormer	BTCV	30	DSC = 0.8335
2022	[165]	UNETR	BTCV	30	DSC = 0.799
2022	[166]	Swin UNETR	BTCV and MSD	30 and 281	DSC = 0.897 and 0.7071
2023	[100]	3D TransUNet	BTCV	30	DSC = 0.8269
2023	[167]	TGPFN	Three private datasets and MSD	313, 53, 50, and 420	DSC = 0.8051, 0.6717, 0.6925, and 0.4386
2018	[168]	Deep LOGISMOS	A private dataset	50	DSC = 0.823±0.078
2020	[169]	Improved UNet based on uncertainty analysis and GCNs	NIH	82	DSC = 0.778±0.063
2020	[170]	DSD-ASPP-Net	NIH	82	DSC = 0.8549±0.0477
2021	[171]	SMCN with Graph-ResNet	A private dataset	661	DSC = 0.738 (PDAC)
2022	[172]	GEPS-Net	NIH	82	DSC = 0.8226±0.0648, IoU = 0.7036±0.0887, HD = 7.88±9.29
2019	[173]	V-NAS	NIH and MSD	82 and 281	DSC = 0.8515 and 0.5886
2021	[174]	DiNTS	MSD	281	DSC = 0.6819, NSD = 0.8608
2023	[175]	SAM	MSD	281	DSC = 0.0547 (box)
2024	[177]	SAM	AbdomenCT-1K	1000	DSC = 0.7686 (box)
2024	[179]	CLIP-Driven Universal Model	MSD	281	DSC = 0.7259, NSD = 0.8976
2021	[181]	MoNet	MSD	281	DSC = 0.74 ± 0.11
2023	[182]	ConDistFL	MSD	281	DSC = 0.5756
2019	[183]	DQN	NIH	82	DSC = 0.8692±0.0492
2021	[184]	Mask-RCNN	NIH	82	DSC = 0.8615±0.0445, IoU = 0.7593±0.646

**Table 6:** Summary of AI methods in CT images for object detection task.

Year	Reference	Model	Dataset	Sample Size	Performance
2020	[185]	Custom pancreatic tumor detection network	A private dataset	2890	Recall = 0.8376, Specificity = 0.9179, Accuracy = 0.9018
2021	[186]	nnDetection	MSD	281	mAP@0.1 = 0.766 (cross validation) and 0.791 (test set)
2023	[188]	RCNN-Crop	NIH	19,000	mAP@0.5 = 0.281
2023	[189]	YCNN	A private dataset	7245	AUC = 1.00, F1-score = 0.99, Accuracy = 1.00

**Table 7:** Summary of AI methods in CT images for prognosis prediction task.

Year	Reference	Model	Dataset	Sample Size	Performance
2020	[160]	CE-ConvLSTM	Three private datasets, MSD and a combined dataset [195]	296, 571, 61, 281 and 90 scans	C-index = 0.651
2021	[190]	RF	A private dataset	98 scans	AUC = 0.84
2022	[191]	Ensemble learning	A private dataset	282 scans	AUC = 0.76 (2-year OS) and 0.74 (1-year recurrence-free survival)
2023	[194]	Custom contrastive learning scheme	A private dataset	157 scans	Accuracy = 0.744, AUC = 0.791, Recall = 0.740, Specificity = 0.750

**Table 8:** Summary of AI techniques in MRIs.

Task	Year	Reference	Model	Dataset	Sample Size	Performance
Classification	2021	[211]	LASSO regression	A private dataset	202	AUC = 0.903
Classification	2018	[212]	PCN-Net	Two private datasets	52 and 68	Accuracy = 0.923
Classification	2018	[214]	ResNet-18	A private dataset	115	Accuracy = 0.91, Precision = 0.86, Recall = 0.99, AUC = 0.90, F1-score = 0.92
Classification	2019	[215]	SVM	A private dataset	139	AUC = 0.78
Classification	2019	[217]	Proportion-SVM	A private dataset	171	Accuracy = 0.8422, Recall = 0.972, Specificity = 0.465
Segmentation	2019	[218]	CNN with Hausdorff-Sine loss function	Two private datasets	180 and 120	DSC = 0.841 and 0.857
Segmentation	2021	[152]	Spiral-ResUNet	Four private datasets	65, 69, 68 and 70	DSC = 0.656, 0.640, 0.645, and 0.653
Segmentation	2020	[219]	Square-window-based CNN	A private dataset	56	DSC = 0.73±0.09
Segmentation	2022	[220]	MMSA-Net	Two private datasets	67 and 67	DSC = 0.6452±0.1953 and 0.6560±0.1532
Segmentation	2023	[221]	MC3DU-Net	A private dataset	158	Precision = 0.75, Recall = 0.80, DSC = 0.80
Segmentation	2016	[222]	CNN with CRF	A private dataset	78	DSC = 0.761
Segmentation	2021	[223]	UDA	Four private datasets	67, 68, 68, and 64	DSC = 0.6138, 0.6111, 0.6190, and 0.6007
Object Detection	2018	[212]	Modified Faster-RCNN	Two private datasets	52 and 68	Precision = 0.589 and 0.598, Recall = 0.873 and 0.889
Prognosis Prediction	2021	[225]	Logistic regression and Cox regression	A private dataset	99	-
Prognosis Prediction	2023	[226]	Cox regression	A private dataset	78	C-index = 0.78

**Table 9:** Summary of AI techniques in EUS images for classification task.

Year	Reference	Model	Dataset	Sample Size	Performance
2022	[231]	SVM and AdaBoost	A private dataset	55	Accuracy = 0.921, Recall = 0.963, Specificity = 0.878
2019	[232]	ResNet-50	A private dataset	3970	Accuracy = 0.940, Recall = 0.957, Specificity = 0.926
2020	[233]	ResNet	Two private datasets	21,406 and 768	DSC = 0.836 and 0.835
2021	[234]	Combination of CNN and LSTM	A private dataset	1350	Accuracy = 0.9826, AUC = 0.98
2021	[235]	ResNet-50	A private dataset	108	Accuracy = 0.8275, AUC = 0.88
2021	[236]	Multi-modal CNN	A private dataset	3575	Accuracy = 0.76, Precision = 0.74, Recall = 0.74, F1-score = 0.74
2022	[237]	Xception	A private dataset	5505	Accuracy = 0.985, Specificity = 0.989, Recall = 0.983, AUC = 1.00
2022	[238]	GoogleNet, ResNet-18, and ResNet-50	A private dataset	66,249	Accuracy = 0.932, Specificity = 0.950, Recall = 0.877, F1-score = 0.870
2023	[239]	ResNet	A private dataset	12,809	Accuracy = 0.9180
2023	[240]	EfficientNetV2-L	A private dataset	22,000	Accuracy = 0.91
2023	[242]	CNNs and ViT models	A private dataset	41	Accuracy = 0.668
2023	[77]	DSMT-Net	LEPset	11,500	Accuracy = 0.877, Precision = 0.842, Recall = 0.801, F1-score = 0.822

**Table 10:** Summary of AI techniques in EUS images for segmentation task.

Year	Reference	Model	Dataset	Sample Size	Performance
2020	[233]	UNet++	Three private datasets	2115, 768, and 28	Accuracy = 0.942, 0.824, and 0.862
2021	[243]	UNet	A private dataset	100	IoU = 0.77
2021	[244]	Attention U-Net	Two private dataset	57 and 364	DSC = 0.794, IoU = 0.741, Accuracy = 0.983, Specificity = 0.991, Recall = 0.797
2022	[245]	DAF-Net	A private dataset	330	DSC = 0.828, IoU = 0.723, AUC = 0.927, Recall = 0.890, Specificity = 0.981, Precision = 0.851
2023	[239]	Attention UNet	A private dataset	1049	DSC = 0.7552, mIOU = 0.6241, Precision = 0.7204, Recall = 0.8003
2023	[246]	UNet++	Two private datasets	4530 and 270	DSC = 0.763, Recall = 0.941, Precision = 0.642, Accuracy = 0.842, mIoU = 0.731

**Table 11:** Summary of AI techniques in EUS images for object detection task.

Year	Reference	Model	Dataset	Sample Size	Performance
2022	[247]	SELSA-TROIA	A private dataset	50	mAP@0.5 = 0.5836
2022	[250]	YOLOv5m	A private dataset	1213	AUC = 0.85, Recall = 0.95, Specificity = 0.75
2023	[251]	Combination of a classifier and YOLO	A private dataset	66,249	IoU = 0.42, Precision = 0.853

**Table 12:** Summary of AI techniques in PET images.

Task	Year	Reference	Model	Dataset	Sample Size	Performance
Classification	2018	[258]	HFB-SVM-RF	A private dataset	1700	Accuracy = 0.965, Recall = 0.952, Specificity = 0.975
Classification	2019	[259]	RBF SVM and Linear SVM	A private dataset	111	Accuracy = 0.85, Specificity = 0.84, Recall = 0.86, AUC = 0.93
Classification	2021	[260]	XGBoost	A private dataset	149	AUC = 0.921
Classification	2023	[262]	TMC	A private dataset	370	Accuracy = 0.75, Recall = 0.77, Specificity = 0.73
Classification	2023	[263]	RAD_model, DL_model, and MF_model	A private dataset	159	Accuracy = 0.901, Specificity = 0.930, Recall = 0.875, AUC = 0.964
Segmentation	2018	[258]	SLIC	A private dataset and NIH	1700 and 82	DSC = 0.789, IoU = 0.654
Segmentation	2023	[262]	UNet with OLP	A private dataset	370	DSC = 0.89
Segmentation	2023	[264]	DenseUNet	A private dataset	48,092	DSC = 0.751
Segmentation	2023	[265]	MFCNet	A private dataset	93	DSC = 0.7620
Segmentation	2024	[266]	CMF module and MIM strategy	A private dataset	93	DSC = 0.7314, IoU = 0.6056, HD = 6.30
Object Detection	2023	[267]	MAFF	A private dataset	880	mAP@0.5 = 0.850
Prognosis Prediction	2023	[268]	NN	A private dataset	58	AUC = 0.830

**Table 13:** Summary of AI techniques in pathological images for classification tasks.

Year	Reference	Model	Dataset	Sample Size	Performance
2022	[274]	PACpAInt	Four private datasets and TCGA	424, 304, 909, 25, and 100	AUC = 0.86 (private test set) and 0.81 (TCGA test set)
2017	[275]	DeepNC	A private dataset	60,036,000	Accuracy = 0.913, Specificity = 0.928, Precision = 0.926, Recall = 0.899
2019	[276]	NLC	TCGA and SEER	190 and 64	AUC = 0.860 and 0.944
2021	[277]	CNN models	A private dataset	138	Accuracy = 0.9561
2022	[278]	CNN with IMSAT	-	-	-
2022	[279]	SI-ViT	A private dataset	5088	Accuracy = 0.9400, Precision = 0.9198, Recall = 0.9068, F1-score = 91.32
2022	[280]	BCNN	A private dataset	3201	Accuracy = 0.7929, Precision = 0.7935, Recall = 0.7933, F1-score = 0.7915
2023	[281]	DACTransNet	TCGA and three private datasets	1336 patches from 190 WSIs, 35, 35, and 38	Accuracy = 0.9634 (TCGA), 0.8973 (Center A), 0.8714 (Center B), and 0.9113 (Center C)

**Table 14:** Summary of AI techniques in pathological images for segmentation task.

Year	Reference	Model	Dataset	Sample Size	Performance
2021	[282]	Modified UNet	A private dataset	16,572	F1-score = 0.86
2021	[283]	SMA block	A private dataset	24	DSC = 0.8347, Precision = 0.8649, Recall = 0.8216
2021	[284]	UNet	A private dataset	231	DSC = 0.8465
2022	[285]	SMANet	A private dataset	165	mDSC = 0.769, mIoU = 0.665
2022	[286]	UNet	A private dataset	5345	F1-score = 0.929
2023	[287]	MLAGG-Net	A private dataset	460	DSC = 0.9002, IoU = 0.8207, Accuracy = 0.9439, Recall = 0.9136
2023	[288]	Multi-task learning framework	A private dataset	555,119	F1-score = 0.97
2024	[289]	Channel-spatial self-attention module	A private dataset	329	DSC = 0.7393, IoU = 0.5942, Accuracy = 0.7526, Precision = 0.8030, Recall = 0.7177

**Table 15:** Summary of AI techniques in multiple modalities analysis.

Year	Modalities	Task	Reference	Method	Dataset	Sample Size	Performance
2021	PET-MRI and CT	Prognosis prediction	[293]	Cox regression	A private dataset	44	AUC = 0.87
2023	CT and MRI	Prognosis prediction	[294]	Cox regression	A private dataset	143	AUC = 0.995, C-index = 0.778
2018	MRI T1w and MRI T2w	Classification	[295]	CNN-based CAD system	A private dataset	139	Accuracy = 0.8280, Specificity = 0.8167, Recall = 0.8355
2018	MRI T1w and MRI T2w	Classification	[214]	PCN-Net	A private dataset	52 and 68	Accuracy = 0.800
2020	MRI ADC, MRI DWI, and MRI T2w	Classification	[296]	Model-driven multimodal deep learning approach	A private dataset	64	Accuracy = 0.736, Specificity = 0.680, Precision = 0.810, Recall = 0.775, AUC = 0.740, F1-score = 0.783
2022	CT and WSI	Prognosis prediction	[297]	ATIIN	A private dataset	356	C-index = 0.70
2023	PET and MRI	Segmentation	[298]	TDSMask R-CNN	A private dataset	71	DSC = 0.7833, Recall = 0.7856, Specificity = 0.9972
2022	CT and MRI	Segmentation	[299]	Improved Res-UNet	A private dataset and MSD	163 and 281	DSC = 0.6416 and 0.5753
2018	CT and MRI	Segmentation	[300]	CNN	Two private dataset	82 and 78	DSC = 0.788 and 0.704
2018	CT and MRI	Segmentation	[301]	CNN-RNN model	NIH and a private dataset	82 and 79	DSC = 0.833 and 0.807, IoU = 0.718 and 0.682, Precision = 0.845 and 0.843, Recall = 0.828 and 0.783
2019	CT and MRI	Segmentation	[302]	Custome 2D/3D method	NIH and two private datasets	82, 216, and 132	DSC = 0.793, 0.796, and 0.816

## Data Availability

The data presented in this study are available in The Cancer Genome Atlas at https://www.cancer.gov/ccg/research/genome-sequencing/tcga (accessed on 15 July 2024), reference number [75]; Medical Segmentation Decathlon at http://medicaldecathlon.com/ (accessed on 15 July 2024), reference number [339]; Surveillance, Epidemiology, and End Results Program at https://seer.cancer.gov/ (accessed on 15 July 2024), reference number [41]; GitHub at https://github.com (accessed on 15 July 2024), reference number [340]; Grand Challenge at https://grand-challenge.org/ (accessed on 15 July 2024), reference number [341]; Synapse at https://www.synapse.org/ (accessed on 15 July 2024), reference number [342]; Zenodo at https://zenodo.org/ (accessed on 15 July 2024), reference number [343]. These data were derived from the following resources available in the public domain: https://academictorrents.com/details/80ecfefcabede760cdbdf63e38986501f7becd49 (accessed on 15 July 2024); https://github.com/JunMa11/AbdomenCT-1K (accessed on 15 July 2024); https://www.synapse.org/Synapse:syn3193805/wiki/89480 (accessed on 15 July 2024); https://github.com/HiLab-git/WORD (accessed on 15 July 2024); https://drive.google.com/drive/folders/1HqEgzS8BV2c7xYNrZdEAnrHk7osJJ–2 (accessed on 15 July 2024); https://zenodo.org/records/4621057 (accessed on 15 July 2024); https://panorama.grand-challenge.org/ (accessed on 15 July 2024); https://doi.org/10.5281/zenodo.8041285 (accessed on 15 July 2024); https://2023paip.grand-challenge.org/ (accessed on 15 July 2024); https://zenodo.org/records/3712669 (accessed on 15 July 2024).
